# Thermomechanics of Picoliter Liquids Encapsulated in Metal Microarchitectures

**DOI:** 10.1002/adma.202515677

**Published:** 2026-02-27

**Authors:** Sung‐Gyu Kang, Kyeongjae Jeong, Bárbara Bellón, Lalith Kumar Bhaskar, Leonardo Shoji Aota, Jeongin Paeng, Dipali Sonawane, Kuan Ding, Se‐Ho Kim, Allison Goetz, Benjamin Apeleo Zubiri, Erdmann Spiecker, Ayman El‐Zoka, Baptiste Gault, Gerhard Dehm, Rajaprakash Ramachandramoorthy

**Affiliations:** ^1^ Max‐Planck‐Institute For Sustainable Materials Düsseldorf Germany; ^2^ Department of Materials Engineering and Convergence Technology Gyeongsang National University Jinju Republic of Korea; ^3^ School of Advanced Materials Science and Engineering Sungkyunkwan University Suwon Republic of Korea; ^4^ Department of Materials Science and Engineering Korea University Seoul Republic of Korea; ^5^ Department of Materials Science and Engineering Institute of Micro‐ and Nanostructure Research and Center for Nanoanalysis and Electron Microscopy (CENEM) Friedrich‐Alexander‐Universitat Erlangen‐Nürnberg IZNF Erlangen Germany; ^6^ Department of Materials Royal School of Mines Imperial College London London UK; ^7^ Department of Materials Engineering KU Leuven Gebroeders De Smetstraat 1 Gent Belgium

**Keywords:** cryogenic temperature, elevated temperature, liquid encapsulation, micromechanics, strain rate

## Abstract

Probing the mechanical behavior of liquids at the nanoscale—especially under hydrostatic stress with various strain rates and extreme temperature conditions—holds significant potential for advancing microfluidic, biomedical, and energy systems. However, it remains experimentally challenging due to the inherent difficulties in encapsulation of liquid at micro/nanoscale and in accurately applying and measuring stress within confined microscale environments. In this work, we present a novel single‐step method for liquid encapsulation at the microscale and subsequent in situ micromechanical testing at extreme dynamic thermomechanical conditions. Localized electrodeposition in the liquid process enables the direct formation of hollow copper microarchitectures containing picoliters of liquid. The presence of the encapsulated liquid was verified via structural analysis at cryogenic and elevated temperatures. We investigated the mechanical role of the confined liquid through compressive tests, demonstrating its incompressibility at room temperature and its enhanced load‐bearing capacity in the ice phase at −160°C. These results reveal enhanced energy dissipation due to the size‐dependent strength of ice. Additionally, we evaluated the tensile response of copper‐ice composites at −160°C using microfabricated push‐to‐pull structures. Our findings outline a new pathway for encapsulation of liquids in metal microarchitectures that could aid and impact fields of microelectronics, pharmaceuticals, and energy storage.

## Graphical abstract

Picoliter‐scale water is encapsulated inside hollow copper microcylinders through a single‐step localized electrodeposition process. Mechanical testing at room and cryogenic temperatures reveals that incompressible liquid and load‐bearing microscale ice enhance the strength and deformation behavior of the composite structure. This work enables direct encapsulation of liquid/ice and probing of their mechanics at the microscale.

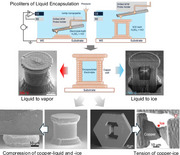

## 1 Introduction

A pursuit of device miniaturization not only pushes the limits of fabricating structural materials but also challenges their assembly with components made from different materials. Accordingly, understanding the mechanical behavior of liquid‐containing composites at the nano‐ and microscale is critically important across a wide range of technologies such as biomedicine [1, 2], energy storage [3, 4], electronics [5, 6], microfluidics [7, 8], and soft robotics [9, 10]. Moreover, this understanding opens up possibilities for probing the structure and deformation behavior of nanoparticles and biological materials dispersed in liquid under various thermomechanical conditions. However, achieving reliable encapsulation of water‐based liquids at the nanoscale and systematically investigating their mechanical responses remains significantly challenging. In particular, the lack of methods capable of forming fully sealed, leak‐free liquid compartments have prevented any direct mechanical interrogation of confined liquids.

To encapsulate the water‐based liquid within solid frames, previous bulk scale studies used multi‐step processes [1, 2, 3, 4, 5, 6, 11]. In pharmaceuticals, for instance, where encapsulating liquid drugs enables localized site‐specific drug delivery via rapid transdermal release into the body requiring emergency care [1, 2], lithography‐based etching is typically used to create drug reservoirs in silicon‐based microchips. These reservoirs are then filled with the liquid drug using micropumps and sealed using subsequent thin‐film fabrication steps [11]. In microscale batteries, essential for powering miniaturized electronics [3, 4], metal electrodes are typically prepared using etching or electroplating and subsequently filled with an electrolyte. Recent advances also include the development of ionic circuits utilizing ions as charge carriers [5, 6]. Jung et al. demonstrated the electrochemical operation of an ionic transistor in an aqueous quinone solution, achieved by packaging a metal electrode and quinone solution at the mesoscale using polydimethylsiloxane (PDMS) [5]. With the above multi‐step processes, the encapsulation of liquid at the nano‐ and microscale is extremely challenging. One‐step encapsulation of liquid within solid frames at such small scales, which would lead to improvements in suggested devices and novel multi‐phase devices, including damage self‐sensing micro‐components, micro/nanoreactors, temperature microsensors, and microactuators, has not been achieved so far.

Liquid encapsulation within solid frames may influence the deformation behavior and micromechanical properties of the entire composite structure. Only limited studies demonstrated that encapsulated liquid gallium enhances the strengths and resilience of millimeter‐sized polymer lattice structures [12, 13]. However, the effect of encapsulating water‐based liquids, essential components for the above‐mentioned applications, on the mechanical properties of metal microarchitectures remains unexplored. It can be hypothesized that encapsulation of water, due to its incompressibility, might increase the strength of metallic microarchitectures and enhance their load‐bearing capability under various loading conditions. Conversely, this increased strength could lead to a catastrophic collapse of the structure during deformation, which can be leveraged in the future as a novel pressure‐relief‐based damage sensing mechanism that activates at a particular strain. Given that actual microscale devices are envisioned to be used in a wide range of combined loading conditions in day‐to‐day and harsh applications, such as avionics/space with strain rates from 0.001 to 1000/s at non‐ambient temperatures, the micromechanical effects of liquid encapsulation and the corresponding deformation behavior of metal microarchitectures under these conditions warrant a detailed investigation.

In this study, we demonstrate for the first time the feasibility of single‐step liquid encapsulation within metal microarchitectures. Hollow copper microcylinders with encapsulated picoliter volumes of liquid were fabricated via a localized electrodeposition in liquid (LEL) process. The LEL printing process is conducted submerged in an acidic liquid medium (pH∼1) that acts as the supporting conductive electrolyte (SCE) solution, containing 0.5 m H_2_SO_4_ + 0.5 mM HCl, to enable a conductive pathway between the three electrodes of the three‐cell electrochemical setup (explained elsewhere in detail [14]). Leveraging this liquid submersion aspect of the LEL manufacturing process, during the voxel‐by‐voxel metal deposition process leaving a void such as a hollow cylindrical wall without any crack spanning across inner and outer surfaces, naturally encapsulates the SCE, and a metal top hat results in a near‐perfect seal to confine the SCE inside without any entrapped air or voids. In situ heating (250°C) and cooling (−180°C) experiments confirmed the presence of the liquid without any leakage owing to the expected phase change (gas and ice, respectively) within the copper microcylinders. We also investigated the impact of the encapsulated liquid on the dynamic mechanical properties of the metal microarchitectures at different temperatures. Both experimental and computational analyses demonstrated that the liquid exerted pressure, leading to increased compressive yield and flow stresses in the copper microcylinders during compression tests at room temperature. At −160°C, which is below the freezing temperature of the liquid SCE, phase transformation to solid ice happened. Furthermore, the size‐dependent strength of the microscale ice was confirmed and attributed to reduced flaw size compared to bulk‐scale ice based on the conventional linear elastic fracture mechanics. Enhanced strength of the microscale ice leads to increased load‐bearing capability and energy dissipation capability of the metal‐ice composite microcylinder. Lastly, we evaluated the load‐bearing capacity of this ice under tensile loading at the microscale, a first‐of‐its‐kind investigation.

## 2 Results and Discussion

### 2.1 Fabrication of Liquid‐Filled Copper Microcylinders

**FIGURE 1:**
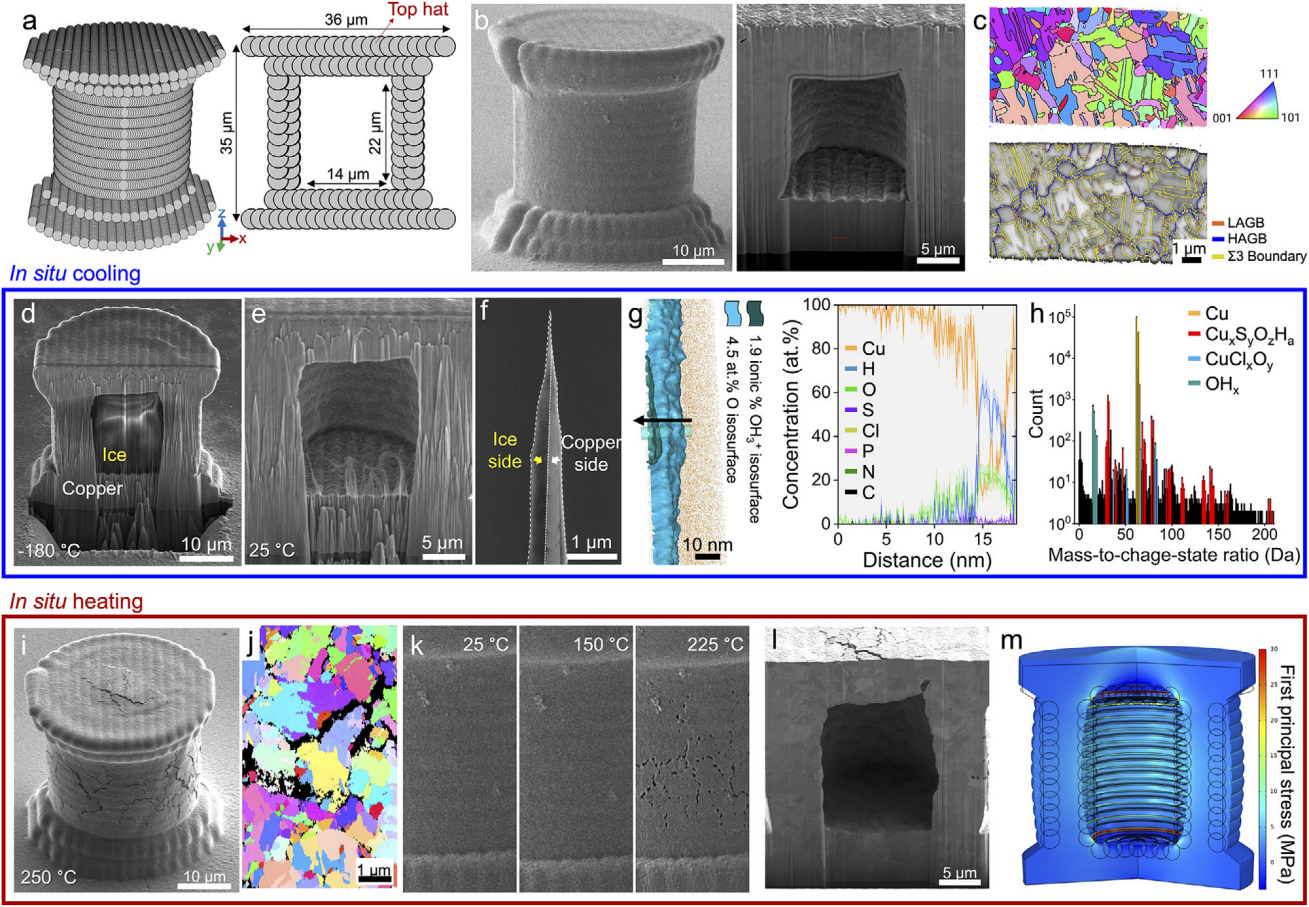
Liquid‐containing copper microcylinder. (a) Exact voxel locations for the LEL process. Left and right images indicate the whole structure and the cross‐section, respectively. (b) SEM image of as‐fabricated copper microcylinder and its cross‐section. (c) Building direction (*z*‐axis) IPF and GB maps obtained from the cross‐section of the microcylinder using EBSD. (d) Cross‐section of microcylinder observed at a cryogenic temperature of ‐180°C. (e) Cross‐section of microcylinder after re‐heating to RT. f) SEM image of the sample prepared for APT analysis. (g,h) APT analysis at the interface between copper and electrolyte. (i) SEM image of microcylinder heated till 225°C. (j) IPF map obtained from the cross‐section of the microcylinder after heating to HT using EBSD. (k) Snapshots of a liquid‐containing cylinder heated to 225°C. (l) Cross‐section of microcylinder after heating to HT. (m) Maximum principal stress distribution in the microcylinder induced by the vapor pressure of the liquid at HT.

Using the LEL process, which deposits micron‐sized metal in a voxel‐by‐voxel manner, we have successfully achieved encapsulation of SCE liquid inside metal microvessels. Figure 1a depicts the exact locations of printing copper voxels for manufacturing a micron‐sized hollow cylinder with encapsulated liquid. The micron‐sized hollow cylinders give an internal cavity of picoliter volume. When the microcylinder is additively deposited from the bottom up, the SCE gets captured within the side walls, and the copper top hat deposition seals it (a step‐by‐step schematic is described in Figure S1). X‐ray absorption contrast images obtained from nano X‐ray computed tomography (nanoCT) analysis in Figure S2 and Movie S1 confirm that the copper microcylinder is fully dense except for the inner cavity for the liquid encapsulation.

The scanning electron microscopy (SEM) images in Figure 1b illustrate the as‐fabricated copper microcylinder and its cross‐section, prepared using focused ion beam (FIB) milling at room temperature (25°C– termed as RT from now). Notably, the cross‐section image reveals no liquid encapsulation, likely due to the instantaneous vaporization of the liquid under the low‐pressure conditions of electron microscopy. It still confirmed a well‐defined central cavity and dense copper casing with a microstructure free of submicron‐sized defects or voids. SEM‐Electron Backscatter Diffraction (SEM‐EBSD) analysis in Figure 1c shows that the electrodeposited copper is polycrystalline with an average grain size of 1 µm and rich in Σ3 twin boundaries, consistent with our previous study [15].

FIB milling was performed at cryogenic temperatures (−180°C) to verify the presence of liquid within the cylinder. Figure 1d displays a cross‐sectional SEM image at cryogenic temperature, where the previously empty space at RT now appears filled with solidified SCE liquid (SCE ice). The volume expansion during the phase transformation of the SCE liquid to ice (typically 9%) led to the permanent deformation of the surrounding copper, resulting in the slightly modified convex shape of the inner cavity. Also, the copper wall, which deformed along with the ice, suggests that there was no leakage during and after the liquid encapsulation. A phenomenon that can be exploited in the future to expand the LEL technique to achieve 4D printing of unique microscale temperature sensors. After reheating the sample to RT, as shown in Figure 1e and Movie S2, the SCE ice in the center vanishes, suggesting the vaporization under increased temperature and low‐pressure conditions analogous to Figure 1b. Accordingly, Figure 1d,e clearly demonstrates successful liquid encapsulation via the LEL process. Cryogenic‐atom probe tomography (cryo‐APT) was used to analyze the chemical composition at the copper‐liquid interface [16, 17]. The copper‐liquid interface was sampled at cryogenic temperature by using a newly developed protocol for lift‐out in the cryo‐FIB [18]. Figure 1f shows the prepared APT needle‐shaped specimen containing an interface between copper and SCE ice, evidenced by the mass contrast. Figure 1g displays a section of the APT reconstruction encompassing the copper and SCE ice interface. The 4.5 at.% O isosurface highlights the Cu‐CuO_x_ interface, indicating a thin surface hydroxide layer (≈6 nm thickness) caused by the encapsulated liquid. In addition to the oxide layer, a thin region with frozen liquid is highlighted by a 1.9 ionic% OH_3_^+^ isosurface. The 1D chemical composition profile indicates OH_3_ in the frozen liquid region between 14–18 nm. When analyzing the bulk chemical composition from this electrolyte region, a 1:2 O/H atomic ratio is observed. Additionally, the presence of OH_3_^+^ and O_2_H_5_^+^ ions as the main peaks in the mass spectrum in Figure 1h is a signature of a water‐based frozen liquid region in this area [19, 20]. Besides water, the SCE is mainly composed of a very low percentage of copper ions with a few other minority ions, such as S, Cl, P, C, and N. Furthermore, during printing, brightener and leveler additives also flow through the hollow AFM tip, thus, organic additives may also be present in the encapsulated liquid.

In the present LEL‐based fabrication route, the encapsulated liquid necessarily shares the chemistry of the supporting conductive electrolyte (0.5 M  H_2_SO_4_ + 0.5 mM HCl). This acidic (pH ∼1) medium provides the ionic conductivity and electrochemical compatibility required for stable electrodeposition; hence, only liquids with similar chemistry can be reliably confined at this stage. In future implementations, the use of alternative deposited metals or electrodeposition chemistries may enable encapsulation of liquids with a broader range of pH and compositions

### 2.2 In situ Heating Based Microreactors

Interestingly, heating the sample above the boiling point of the liquid leads to a significant structural change in the copper microcylinder. The SEM images in Figure 1i show surface cracking of the cylinder when heated to 250°C. SEM‐EBSD analysis in Figure 1j reveals intergranular cracking at the copper grain boundaries. Snapshots in Figure 1k captured during the in situ SEM heating experiment display the cracks emerging when the temperature reached 225°C. Serial cross‐sections of the heated microcylinder displayed in Movie S3 confirm that the intergranular cracking on the surface extends to the inside of the copper wall. The grain growth of copper, which typically occurs at above 400°C can lead to intergranular fracture‐like features [21]. Grain growth can be excluded from possible mechanisms by conducting an in situ SEM heating experiment of the microcylinder with the same inner cavity but without encapsulated SCE. This unfilled cylinder has a special micropore channel connecting the inner cavity to the outer surface (Figure S3). When the printed substrate with the microcylinder is removed from the printing chamber, the SCE liquid inside the microcylinder drains out automatically through the micropore channel, leaving the microcylinder unfilled. If any SCE liquid remains, it can further evaporate in the electron microscope chamber owing to the high vacuum environment before heating. SEM image of the unfilled cylinder, without liquid, after heating to 250°C doesn't exhibit cracks or crack‐like features on the surface (Figure S3e,f), suggesting that the cracks seen in the liquid filled cylinders after heating are not possibly due to the grain growth of copper. Moreover, the cross‐section of the heated liquid‐filled cylinder in Figure 1l shows that the inner wall was deformed during heating, and presumably, this can be attributed to the phase transition of SCE liquid to a gaseous state.

In our study, we hypothesize that the brittle‐like intergranular fracture is caused by stress corrosion cracking (SCC) in copper walls. The vapor pressure exerted by the evaporated SCE liquid at high temperatures may develop stresses in the copper walls comparable to the critical SCC stress. The vapor pressure can be estimated using Tetens’ equation below:

(1) $$P=0.61078\exp\left(\frac{17.27T}{T+237.3}\right)$$

where *T* is the temperature in degrees Celsius. It suggests that the vapor pressure of SCE liquid at 225°C is 4 MPa. Further, Finite Element (FE) analysis in Figure 1m indicates that the tensile stress field developed by the vapor pressure of the gaseous SCE, especially in the corners and grooves (the result of the layer‐by‐layer printing process), reaches a maximum of 30 MPa. For copper, the critical stress for SCC is known to be around 20 MPa [22]. Yet another important factor that supplements the stress on the copper walls is the expected dissociation of sulphuric acid in the SCE at temperatures of ≈225°C, producing sulphur trioxide gas [23].

Notable previous studies on SCC behaviors of copper intergranular fracture identify oxide rupture [24] and tarnish rupture [25] as possible mechanisms. Yet such mechanisms necessitate the formation of a protective copper oxide film. While we do observe a copper hydroxide film using cryo‐APT, we anticipate that the environmental conditions for the continuous formation of a protective copper oxide film along copper inner walls or copper cracks are not met in our case. This is due to the highly acidic nature of the SCE (pH∼ 1) and the presence of chloride [26]. The semi‐protective copper hydroxide film is expected to further dissolve with increasing temperatures, due to an increase in acid dissociation and overall acidity of the SCE. APT results in Figure S4 show that there are oxygen and chlorine segregations in the copper inner wall only from the heated cylinder, indicating the overall corrosion of copper up to the point of fracture. As such, these unique acidic SCE liquid‐filled copper microcylinders are expected to be used as microreactors and enable controlled corrosion explorations. Future studies to confirm the exact mechanism of SCC failure in such copper‐liquid (acidic) microreactors will target the copper microstructure‐dependent evolution of the solid‐liquid interface at different temperatures, using cryo‐APT to examine the changes in copper oxidation state at the wall surfaces, and the exact roles of sulphate and chloride ions. For now, we can hypothesize intergranular fracture mode as the responsible mechanism for the accelerated corrosion of copper atoms along grain boundaries in the microcrystalline copper cylinder studied in the current work, as reported in various previous studies [26, 27, 28].

### 2.3 Liquid‐Filled Microcylinder: Rate‐Dependent Liquid Escape at Room Temperature

We explored the deformation behavior of liquid‐filled microcylinders at RT by conducting in situ compression tests with a piezoelectric‐actuator‐based micromechanical tester inside an SEM. To discern the liquid's effect, we also conducted the same compression tests on the unfilled cylinder.

**FIGURE 2:**
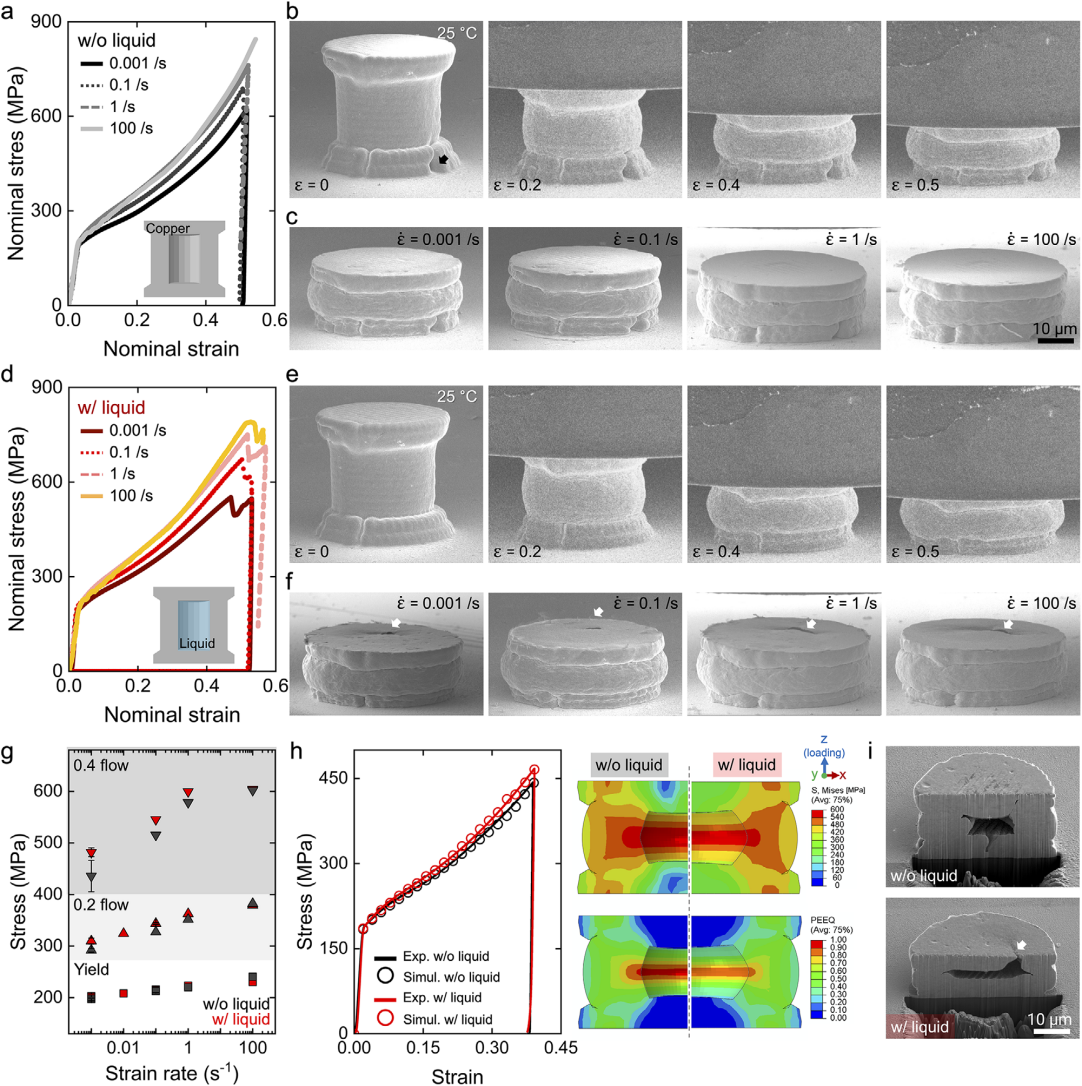
Compression test of copper microcylinder at room temperature. (a) Stress‐strain curve of unfilled cylinders. (b) Snapshots of the unfilled cylinder during compression at 0.001/s. (c) Post‐deformation SEM images of an unfilled cylinder compressed to ε  =  0.5 at different strain rates. (d) Stress‐strain curve of liquid‐filled cylinders. (e) Snapshots of a liquid‐filled cylinder during compression at 0.001/s. (f) Post‐deformation SEM images of a liquid‐filled cylinder compressed to ε  =  0.5 at different strain rates. (g) Yield and flow stresses of cylinders. (h) Finite element analysis of the deformation of liquid‐filled and unfilled cylinders compressed at 0.001/s. (i) Cross‐sections of liquid‐filled and unfilled cylinders after compression at 0.001/s.

Figure 2a–c presents the in situ compression test results for the unfilled cylinder at RT under various strain rates (0.001, 0.1, 1, and 100/s). The nominal stress‐strain curves in Figure 2a commonly show a linear stiff increase of stress to strain of 0.025 corresponding to the elastic deformation, and a subsequent gradual increase of stress by further deformation corresponding to the plastic deformation. Notably, the stress‐strain curve at 100/s is reported without the unloading segment, due to the load cell ringing during the unloading process [29]. In terms of elastic deformation, on the one hand, the stress‐strain curves at all strain rates are almost overlapping each other. On the other hand, during plastic deformation, the stress level increases with the strain rate. Snapshots of the unfilled cylinder compressed at 0.001/s in Figure 2b confirm gradual sidewall deformation, with no abrupt changes (the corresponding video is displayed in Movie S4). A black arrow indicates the pore channel connected to the cylinder's outer bottom surface. Deformed shapes in Figure 2c show no noticeable difference with respect to the strain rate from 0.001 to 100/s, suggesting that the strain rate dependency of compressive strength originates from the intrinsic mechanical properties of copper. Our previous study on the micromechanical properties of copper micropillars fabricated by the same deposition process confirmed the strain rate sensitivity of copper, and it is consistent with the current study [15].

Figure 2d–f displays the in situ compression test results for the liquid‐filled cylinder under the same conditions. Analogous to the unfilled cylinder, the nominal stress‐strain curves in Figure 2d reveal that the mechanical response of the cylinder during elastic deformation is almost constant across strain rates, whereas the flow stress increases with strain rate. Notably, there is a stress drop in every stress‐strain curve. This event can be attributed to an escape of SCE liquid during compression. Figure 2e confirms gradual sidewall deformation in the cylinder compressed at 0.001/s, with no abrupt changes (corresponding video is displayed in Movie S5). However, as Figure 2f indicates, the top surfaces of the compressed cylinders exhibit holes (highlighted by white arrows) after compression, suggesting SCE liquid escape during compression at high strain. Figure S5 shows that a strain where the stress drop occurs increases with the strain rate. Typically, with increasing strain rate, the material becomes stronger, meaning higher strain is required for the liquid to escape. Figure 2g directly compares the yield and flow stresses of liquid‐filled and unfilled cylinders relative to strain rate. Both cylinders show clear rate sensitivity in yield and flow strengths as described above. However, the rate sensitivity appears independent of the liquid's presence, with only a marginal stress increase due to the liquid.

Based on the Gibson and Ashby framework, how the filling of the inner cavity influences the effective mechanical response of a porous solid can be elucidated. The effective strength of a porous solid typically scales with its relative density as σ*/σ_*s*_∝(ρ*/ρ_*s*_)^*n*^, where the exponent *n* depends on the dominant deformation mode. Generally, a lower *n* corresponds to stretch‐dominated (tensile) deformation, while a higher *n* reflects bending‐dominated behavior. From the yield strengths of microcylinders in this study and micropillars from our previous study, *n* for microcylinders compressed at 0.001 s^−^^1^ is evaluated. At RT, the unfilled cylinder exhibited *n* of 1.94, corresponding to a mixed bending–stretching shell response. When filled with SCE, the microcylinder exhibits a slightly lower *n* of 1.85, which can be attributed to the incompressibility of SCE.

FE simulation describes the deformation behavior of microcylinders and also supports the estimated *n* values. A constructed model employed the Johnson–Cook plasticity model for copper and assumed encapsulated water as a nearly incompressible medium. The hardening parameters of copper (*A, B, n*) and the thermal softening exponent (*m*) were calibrated through a genetic‐algorithm‐based optimization procedure using the experimental load–depth data from the empty copper cylinders tested at 0.001/s for each temperature. Figure S6 shows the compression load‐displacement curves of unfilled cylinders at RT and two different strain rates (0.001 and 0.1/s). Clearly, the constructed FE model well‐described the compressive behavior of unfilled cylinders. Figure 2h shows the stress‐strain curves of unfilled and filled cylinders at RT and 0.001/s. These simulations also indicate a slight increase in yield and flow stresses in the liquid‐filled cylinders, attributed to pressure from the encapsulated liquid. The simulation also suggests that the deformed shape of the inner wall differs with and without the existence of the liquid. It can be deduced that the incompressible liquid hinders the inward deflection and reduces the stress and strain concentrations in the copper wall during compression. As a result, the top plate of the liquid‐filled cylinders shows higher plastic strain (stretch‐dominated) than that of unfilled cylinders. Experimentally measured top plate thickness in Figure S7 confirms that larger plastic deformation occurred in the top plate of the liquid‐filled cylinder than in the unfilled cylinder. In Figure 2i, the cross‐section SEM images of the compressed unfilled (top) and liquid‐filled (bottom) cylinders agree well with the FE analysis. Compared to the unfilled cylinder, the liquid‐filled cylinder shows relatively less inward deflection of the wall, and importantly, there is an opening of the top plate after the compression. This opening specifically occurred at a printing defect in the top hat, which was likely caused by flow disturbances of the SCE. During sealing of the cylinder, the SCE may have flowed opposite to the ink flow, resulting in a few skipped voxels (see Figure S8 for details). This defect subsequently served as a pathway for the encapsulated liquid to escape the cylinder during compression (white arrow in Figure 2i), and the moment of escape was captured as a stress drop in the stress‐strain curves in Figure 2d. Future studies will focus on optimizing the printing process to control the shape and size of such stress concentrations, for example, a 3D notch design. This will allow, for the first time, the fabrication of precise rate‐dependent microscale strain sensors.

### 2.4 Cryomechanics of Liquid‐Filled Microcylinder: Phase Transformation to Ice

**FIGURE 3:**
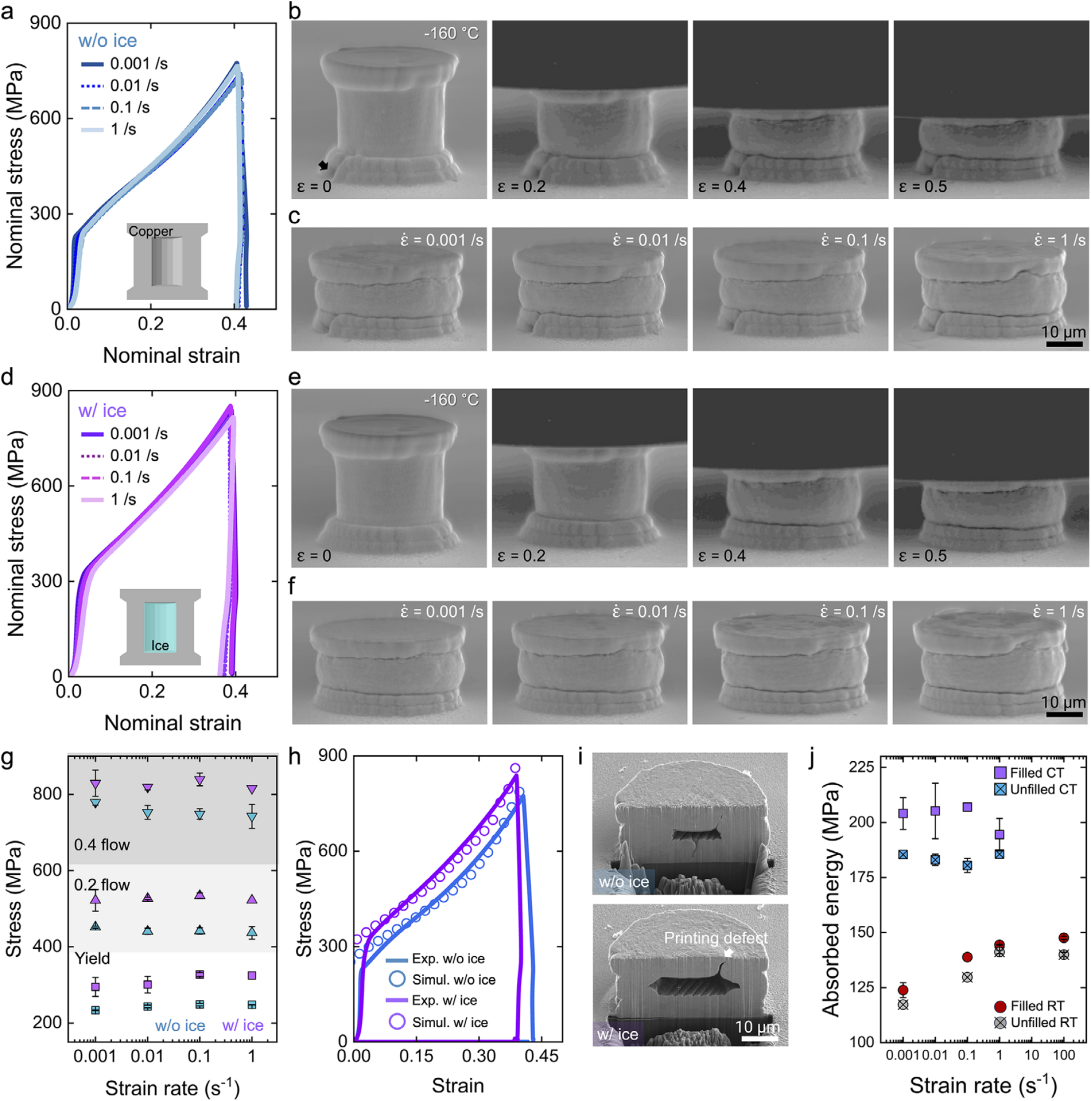
Compression test of copper microcylinder at cryogenic temperature of −160°C. (a) Stress‐strain curve of unfilled cylinders. (b) Snapshots of the unfilled cylinder during compression at 0.001/s. (c) SEM images of an unfilled cylinder compressed to ε  =  0.4 at various strain rates. (d) Stress‐strain curve of ice‐filled cylinders. (e) Snapshots of ice‐filled cylinder during compression at 0.001/s. (f) SEM images ice‐filled cylinder compressed to ε  =  0.4 at various strain rates. (g) Comparison of yield and flow stresses of cylinders. (h) Finite element simulations comparing deformation profiles of ice‐filled and unfilled cylinders compressed at 0.001/s. (i) Cross‐sections of ice‐filled and unfilled cylinders after compression at 0.001/s. (j) Absorbed energy of microcylinders compressed at RT and CT.

In order to explore the effect of water‐to‐ice phase change on the copper/liquid composite properties, we further investigated their deformation behavior at cryogenic temperatures (−160°C – termed as CT from now). In situ compression tests were conducted with the same micromechanical tester with necessary modifications that allow localized cooling of the sample and tip (see methods section). A temperature profile obtained from thermocouples attached to the sample in Figure S9 indicates that the maximum cooling rate of −0.3°C/s was achieved. Due to limitations in the mechanical testing setup, only strain rates of 0.001, 0.01, 0.1, and 1/s were achievable. Compression tests of the unfilled cylinder without liquid at CT under various strain rates are shown in Figure 3a–c. The nominal stress‐strain curves in Figure 3a at all strain rates are approximately overlapping with each other, indicating that there is negligible strain rate sensitivity at CT. This is consistent with our previous study on copper micropillars compressed at CT, which also showed similar rate insensitivity in yield and flow strengths ^15^. Snapshots of the cylinder compressed at 0.001/s in Figure 3b show gradual deformation, which is analogous to that observed at RT (the corresponding video is displayed in Movie S6). The compressed shapes in Figure 3c confirm that no noticeable change in the deformed morphology occurs as a function of the strain rate.

Figure 3d–f displays the in situ compression test results for the liquid‐filled cylinder at CT and various strain rates. The nominal stress‐strain curves in Figure 3d indicate again that the liquid‐filled cylinders exhibit negligible strain rate sensitivity, but noticeably increased yield and flow strengths compared to those of unfilled cylinders. The increased strengths at CT can be attributed to the SCE liquid inside the cylinder phase transforming to solid ice. Furthermore, the liquid‐filled cylinder at CT didn't show any stress drop during compression, indicating that there was no leakage or fracture of the cylinder. Snapshots of the cylinder compressed at 0.001/s in Figure 3e show no crack or fracture occurring at the side wall (the corresponding video is displayed in Movie S7). The compressed shapes of cylinders in Figure 3f show flat top plates without any holes observed when the liquid‐filled cylinders are compressed at CT.

Figure 3g shows a comparison of the yield and flow strengths of liquid‐filled and unfilled cylinders compressed at CT relative to strain rate. As confirmed above, both these cylinders show negligible sensitivity in yield and flow strengths. However, it is clear that the yield and flow strengths increase due to the existence of the SCE ice in the liquid‐filled microcylinder. A load‐bearing capability of the SCE ice can be directly extracted from the compressive load‐depth curves based on force balance as below:

(2) $$\sigma_{ice}=\sigma_{filled}\;\frac{A_{cyl}}{A_{ice}}-\sigma_{unfilled}\frac{A_{cyl}}{A_{ice}}$$

where σ_*filled*_ and σ_*unfilled*_ are nominal yield stresses of filled and the unfilled cylinders compressed at CT shown in Figure 3g, respectively. *A_cyl_* and *A_ice_* are the cross‐sectional area of the microcylinder and the SCE ice normal to the loading direction. The averaged σ_*ice*_, the load bearing capapility of the SCE ice, at nominal strain from 0.05 to 0.4 was calculated as ≈470 MPa. Mechanical properties can also be extracted by finding the best FE solution matching the stress‐strain curves from the experiment (Figure 3h). With this approach, the elastic modulus and the compressive strength of the SCE ice are identified as 9 GPa and 300 MPa, respectively, which are comparable to the compressive strength from the load‐bearing capability analysis. Considering that the chemical composition of the encapsulated liquid is mostly water, the elastic modulus and the compressive strength of the ice were referred from the literature to be 9 GPa and 40 MPa, respectively [30, 31]. It should be noted that the known mechanical properties of the ice are obtained at the bulk scale. Given that the volume of the encapsulated liquid is pico‐liters, such a high compressive strength between 300–470 MPa could possibly be attributed to the small dimensions of ice and its microstructure, which requires further investigation [30]. Linear elastic fracture mechanics can rationalize the size‐dependent strength observed experimentally and computationally. Assuming size‐independent *K_IC_* and a characteristic flaw size *a*, the fracture/critical stress scales as σ∝*a* ^−1/2^. Reducing the representative flaw from bulk‐scale values (e.g., *a_macro_* ≈100 µm) to confined picoliter volumes (estimated *a_micro_* ≈1−10 µm, based on cross‐sectional SEM image in Figure 1d) yields a strength amplification factor of ((*a_macro_*/*a_micro_*)^1/2^ ≈ 3−10), which predicts 120–400 MPa—in good agreement with our experiment‐ and FE‐derived values. This supports the size‐dependent strength interpretation and explains the enhanced load‐bearing and energy absorption observed for the metal–ice composite microcylinders.

Exponent *n* for dominant deformation mode is evaluated from compressive yield strengths of microcylinders at CT and at 0.001 s^−^^1^. The unfilled cylinders showed *n* of 2.41. In contrast, the liquid‐filled cylinders exhibited *n* of 1.86, as the ice core shared load. Detailed deformation behavior is described via FE simulation as follows. Notably, the exponent *n* of liquid‐filled cylinders at CT and RT is almost the same. This similarity indicates that during the onset of plastic deformation, both the encapsulated liquid and solid ice induce a comparable degree of stretching in the cylinder top plate. Beyond yielding, however, their roles diverge: the incompressible liquid contributes to the load‐bearing capability of the microcylinder but, at higher strain, its incompressibility drives local hole formation at the top plate. In contrast, the solid ice undergoes plastic deformation and continues to share load with the copper wall.

Stress and plastic strain distribution maps in Figure S10 also suggest that the SCE ice hinders inward deflection of the side wall during compression and reduces stress and strain concentration, emphasizing its load‐bearing capability. The room‐temperature FIB‐based cross‐sectional SEM images of both the liquid‐filled and unfilled cylinders after compression at CT, in Figure 3i are almost identical to the compressed shapes predicted by FE analysis. Note that there is no opening of the top plate as the SCE ice would not escape the cylinder during compression. Instead, high stress on the top plate, due to the interface between copper and the SCE ice restricting deformation, leads to plastic deformation with higher plastic strain at the top plate in the filled cylinder compared to the unfilled cylinder at CT. A thickness measurement of top plates after compression in Figure S11 indicates that the SCE ice leads to a larger thickness reduction of the top plate for the liquid‐filled microcylinders than for the unfilled microcylinders.

Combined with copper's intrinsic rate‐ and temperature‐dependent strength, encapsulated liquid leads to enhanced energy dissipation capability of the microcylinder under compression. The energy dissipation capability of unfilled and liquid‐filled microcylinders was directly evaluated from stress‐strain curves. Considering the maximum compressive strain of cylinders at CT (about 0.4), we compared the energy dissipation capability of every cylinder compressed at various temperatures and strain rates up to this strain. The absorbed energy plot in Figure 3j clearly shows that unfilled cylinders at RT show rate sensitivity resembling the strength plot in Figure 2g. At RT, encapsulated SCE leads to a 5% increased absorbed energy of the microcylinder under compression, which can be attributed to the incompressibility of the liquid. Unfilled cylinders at CT show a negligible rate‐independent absorbed energy, which is consistent with the strength plot in Figure 3g. Importantly, at CT, SCE ice leads to a 12% increased absorbed energy of the microcylinder under compression, possibly originating from the size‐dependent strength of ice. It further highlights the mechanical robustness of ice‐containing metallic microarchitecture suitable for future extreme applications, accompanying high strain rates and cryogenic temperatures.

### 2.5 Tensile Property: Copper‐Ice Core‐Shell Microarchitectures

**FIGURE 4:**
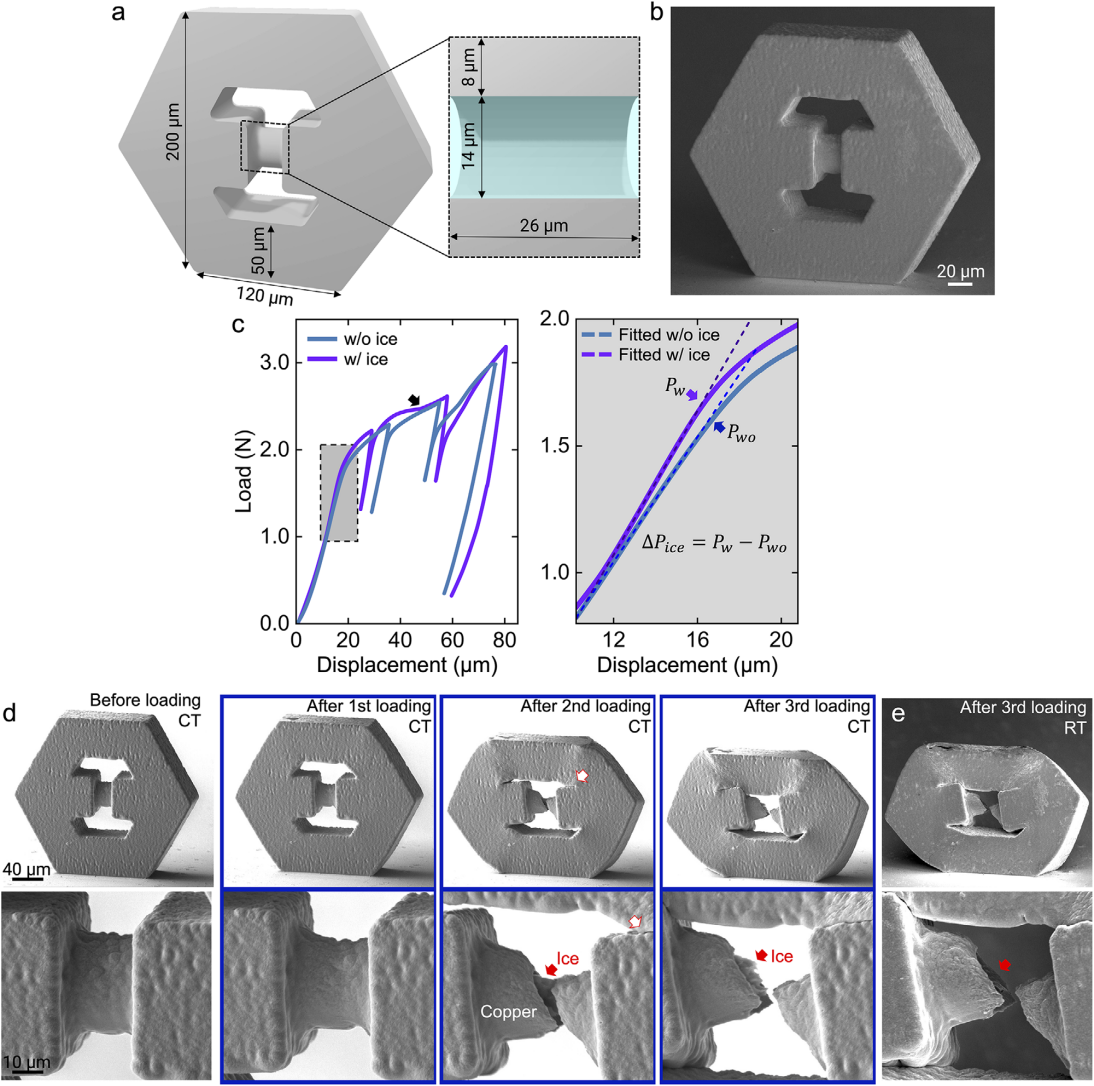
Compression test of copper liquid‐containing push‐to‐pull structure at cryogenic temperature of ‐160°C. (a) Computer‐aided design of the structure. (b) SEM image of as‐fabricated copper push‐to‐pull structure. (c) Load‐displacement curve of push‐to‐pull structure with and without encapsulated ice. (d) Snapshots of push‐to‐pull structure during compression.

Given the remarkable increase in compressive strength of SCE ice at small scales, we also explored its tensile properties by subjecting a push‐to‐pull copper microarchitecture—featuring a liquid‐filled microcylinder—to tensile loading. Figure 4a shows the push‐to‐pull structure with a liquid‐containing section. This design includes a solid 50‐µm‐thick hexagonal copper frame to ensure uniform tensile deformation at the relatively thin liquid‐containing section (an 8‐µm‐thick copper wall surrounding a 14‐µm‐thick liquid/ice core). Bauer et al. demonstrated that pushing down the top plate of such a 3‐D push‐to‐pull structure leads to axial stretching of the gauge section [32]. Figure 4b shows an SEM image of the as‐fabricated push‐to‐pull structure, consistent with the original design. The left plot of Figure 4c presents the load‐depth curve of this structure at CT obtained by pushing down the top plate with a flat punch indenter (the corresponding in situ video is shown in Movie S8). The temperature condition was the same to compression tests of microcylinders to roughly compare the strengthening effects of microscale ice subjected to compression and tension. Due to the maximum displacement limit based on the piezoactuator's range used in the testing system, multiple loading and unloading cycles were required to induce a fracture at the SCE ice‐containing section. Figure 4d shows SEM images of the structure before and at the end of each loading cycle. The SCE ice‐containing section shows plastic deformation under tension by first loading. The corresponding FE model further confirms that compression of the push‐to‐pull structure generates pronounced plastic strain localization in the ice‐containing section (Figure S12). After the second loading, the copper walls of the SCE ice‐containing section fractured under tension, exposing the ice (indicated by a red arrow). Notably, the corresponding load‐displacement curve shows an increase in slope during the second loading (displacement of 60 µm, black arrow in Figure 4c). This is due to the early contact between the solid hexagonal frames (white arrow with red line in Figure 4d). During the third loading, plastic deformation began at a lower load than during the second loading (Figure 4c), which can be attributed to the distortion of the frames with large deformation. The corresponding SEM image indicates a distorted top plate after the third loading. The post‐test SEM image clearly shows fractured SCE ice debris (Figure 4d). Finally, heating the sample to RT leads to the melting of SCE ice, and the liquid instantaneously evaporates into the SEM chamber, owing to the high vacuum environment (Figure 4e; Movie S9). This proved that under similar mechanical loadings at RT in ambient pressure conditions, the liquid inside will leak, and using fluorescence dye additions to the liquid, this method can be leveraged for quick damage sensing in micro/nanoscale applications.

To quantify the ice's strengthening effect, we fabricated a push‐to‐pull structure with the same geometry but without encapsulated SCE ice. The corresponding FE model also confirms plastic strain is intensively developed at the hollow gauge section during loading (Figure S12). It was achieved by a small hole (diameter of 1.5 µm) on the surface of the liquid‐containing section via FIB milling (Figure S13). It should be noted that during axial stretching of the gauge section, the hole at the side surface could locally increase the stress by acting as a stress concentrator (stress concentration factor is approximately 3). It suggests that early yielding may occur around the hole, leading to overestimation of the ice's strengthening effect. However, as shown in Figure 1b and Figure S14, the voxel‐by‐voxel printing sequence can also generate notch‐like printing defects at the inner wall surface. Savruk et al. calculated stress concentration factors of half planes with various curvilinear edges [33]. It gives an approximate stress concentration factor of 2.6, slightly lower than that of a hole at the outer surface. Accordingly, although early yielding around the hole is likely, the difference in yield load between the hole‐containing structure and the reference structure without a hole and liquid (which is not possible to fabricate in this study) is expected to be negligible.

The stress‐strain curve of this structure is indicated as the blue curve in the left plot of Figure 4c. Corresponding SEM images in Figure S13 after each loading segment indicate that the push‐to‐pull structure without encapsulated ice also underwent early frame contact‐induced hardening during the second loading. Furthermore, the hollow copper walls fractured under tension at the end of the second loading. It suggests that the ice's load bearing capability can be quantitatively estimated by comparing load levels during the first loading of the push‐to‐pull structures with and without encapsulated SCE ice. The right plot of Figure 4c presents the difference in load levels during the first loading, with highlighted loads for the yield of each structure (*P_w_* and *P_wo_* for structures with and without encapsulated SCE ice, respectively. The difference in yield load (Δ*P_ice_*) is about 30 mN. Considering the cross‐sectional area of ice, load bearing capability, or strengthening effect of ice (σ_*Ice*_) under tensile loading can be calculated as ≈195 MPa. The axisymmetric gauge‐only model with rough interface (induced by notch‐like printing defects at the inner wall surface) confirms that the strengthening effect of ice can be attributed to the friction between ice and copper, and also the roughness (Figure S15). At sufficiently low temperatures (below approximately −80°C), the friction coefficient µ between ice and metal increases dramatically, reaching values in the range µ ≈ 0.3–0.8 due to enhanced solid–solid interlocking and adhesion. In the present simulations, we deliberately selected the lower bound of this reported range (µ = 0.3) to avoid overestimating the mechanical contribution of ice and to provide a conservative assessment of its load‐bearing effect. The model with the smooth interface estimates a negligible strengthening effect of ice (ΔL ≈ 5 mN), suggesting limited tensile stress is transferred to the ice even in the presence of friction. However, the model with a rough interface estimates a noticeable strengthening effect of ice, quite close to the experimentally measured value (ΔL ≈25 mN). It suggests that geometric interlocking combined with friction produces a constraint, leading to effective stress transfer to the ice. This result indicates that the experimentally observed strengthening is mechanically consistent with load sharing by confined ice, while also highlighting that the magnitude depends on the roughness of the copper–ice interface. Accordingly, a further increase in interfacial roughness is expected to enhance the strengthening effect by promoting greater mechanical interlocking.

To experimentally verify the interfacial characteristics between ice and copper, more refined copper‐ice geometries to quantitatively separate the ice's strength and the interfacial behavior need to be designed in future studies.

As a perspective, in the future, liquids other than the SCE can be used to fill the voids in the metal microcylinders. Some examples would include: liquids immiscible with the SCE that can be introduced into the voids using dispersion from the LEL cantilever and modified SCE liquid with different compositions and pH levels to conduct a variety of microreactor‐based corrosion explorations. The LEL method can also be used to deposit other metal casings, including nickel and gold, to encapsulate the liquids. The current study sets the precedence for using LEL printing‐based pathways to fabricate and investigate even more complex metal‐liquid multiphase microarchitectures, such as microlattices, in the future.

## 3 Summary

In summary, this study demonstrates a unique methodology to encapsulate liquids in microscale metal vessels using the localized electrodeposition in liquid process for the first time. We confirmed the presence of liquid in the microstructures at both cryogenic temperatures and high temperatures through detailed surface and cross‐sectional structural characterization. Furthermore, we systematically investigated the impact of liquid presence on the micromechanical properties of metal microarchitectures. At room temperature, the pressure exerted by the encapsulated liquid resulted in a nominal increase in yield and flow stresses. Further, rate‐dependent leakage at different strains of the encapsulated liquid was observed. At cryogenic temperatures, the load‐bearing capacity of ice formed from the SCE liquid contributed to significant increases in yield and flow stresses and energy dissipation capability. Additionally, the push‐to‐pull microarchitectures containing liquid were used to investigate the tensile properties of solidified SCE‐based microscale ice when cooled to cryogenic temperatures. The well‐defined cavity shape and dense microstructure of these architectures enabled precise observation and explanation of the effects of both liquid and ice at the microscale. The novel liquid encapsulation method in microscale and its distinct effects on the deformation behavior of metal microarchitectures establish a versatile platform for probing the coupled thermomechanical response of confined liquids and phase‐changing media. It can be further utilized in future micro‐ and nanoreactors, temperature‐based actuators and sensors, and microscale damage inspection.

## 4 Experimental Section

### 4.1 Printing of Microarchitectures

The copper microarchitectures were fabricated employing the LEL process, utilizing the force‐controlled electrodeposition capabilities of the CERES system (Exaddon AG, Switzerland). Copper ions (0.5 m CuSO_4_ in 51 mM H_2_SO_4_ and 0.48 mM HCl with brightener and leveler additives) flow through a 300‐nm‐diameter orifice in a hollow silicon nitride AFM tip immersed in a standard three‐cell electrochemical cell by applied pressure. Deposition of the reduced ions as metal droplets occurred on a Si/Ti (10 nm)/Cu (100 nm) substrate connected to the working electrode, with precise placement at designated locations. The deposition process follows a voxel‐by‐voxel methodology. Based on a laser deflection signal on top of the AFM cantilever, the cantilever was moved to subsequent voxel coordinates as each growing copper voxel causes a deflection. This deflection threshold was maintained in the order of 1 nm. The LEL process parameters were optimized for different structures.

For the microcylinder, a voxel spacing of 0.5 µm was used; therefore, the voxel diameter was set to approximately 3.8 µm to ensure sufficient overlap and a smooth wall surface. For the push‐to‐pull structure, a larger voxel spacing of 1 µm was employed to obtain a broader gauge section, requiring a voxel diameter of approximately 5.0 µm. To identify the optimal deposition pressure, the applied potential was fixed at −5 V, and the pressure was varied from 20 mbar to 100 mbar while analyzing the resulting voxel diameter and morphology. The experiments revealed that a pressure of 60 mbar produced a voxel diameter of 3.90 µm, which was the optimal parameter for microcylinder fabrication, whereas 100 mbar yielded a voxel diameter of 4.98 µm, which was the near‐ideal printing pressure for the push‐to‐pull architecture.

Consistent with previous studies, the copper structures fabricated via LEL exhibited dense microstructures and smooth surfaces [34]. The LEL printing process was conducted submerged in a supporting conductive electrolyte (SCE) solution. Detailed methodology and additional information on the LEL process was comprehensively described in prior publications [14]. Step‐by‐step schematics of the fabrication of microcylinders with and without SCE liquid was described in Figures S1 and S3.

### 4.2 Atom Probe Tomography (APT)

With both the sample stage and the manipulator under cryogenic temperatures (−190°C and −175°C, respectively), a 25 µm bar lift‐out was taken from the middle of a single copper cylinder following the chromium redeposition method in a Helios 5 CX SEM‐FIB (focused ion beam) with Ga ion source [35]. Due to the cryogenic temperatures, the use of the usual gas injection system was hindered during the welding of the samples to the Si coupon, used as support for the samples. Thus, a chromium target was in situ sputtered into the junction between the samples and the silicon coupon, where it will deposit and create a stable junction. Each sample was sharpened in the Ga‐FIB, aiming at the liquid/solid interface. All samples were transferred from the FIB to the Cameca LEAP (local electrode atom probe) 5000XR under HV (high vacuum, < 10^−6^ Pa) and cryogenic temperature (−196°C) in a Ferrovac suitcase. APT measurements were performed at a constant base temperature of 50 K, a laser pulse energy, and a rate of 80 pJ and 125 kHz, respectively. The target detection rate was 1% (1 evaporative event every 100 pulses). The reconstruction was performed in the software AP Suite 6.3, following the voltage protocol.

### 4.3 Internal Characterization of the Wall Structure Using nanoCT

The internal structure of the walls was investigated utilizing X‐ray nano‐computed tomography (nanoCT) via the lab‐based ZEISS Xradia 810 Ultra X‐ray microscope, equipped with a 5.4 keV (Cr Kα) rotating anode source. Due to copper's strong X‐ray attenuation, the absorption contrast was utilized. Further, the samples were investigated in the large‐field‐of‐view mode of the microscope, allowing for a resolution of down to 150 nm in a field of view of (64 µm)^3^ and a pixel size of 65 nm. The exposure time was set to 90s, and the number of projections at 661 or 1601, depending on the sample. The resulting tilt series were reconstructed via an implementation of the simultaneous iterative reconstruction technique (SIRT) based on the ASTRA toolbox^1,2^, choosing 100 iterations for these samples. The reconstructed volumes were visualized and analyzed with the software arivis Vision 4D 4.1.0.

### 4.4 In Situ Micromechanical Test

Compression tests were conducted in situ using an Alemnis indenter (Alemnis AG, Switzerland). These experiments at room temperature and cryogenic temperature utilized the Alemnis Standard Assembly (ASA) and the Alemnis LTM‐Cryo, respectively. The indenter was integrated into a Zeiss Gemini 500 Scanning Electron Microscope (SEM) (Zeiss, Germany) and a JEOL JSM‐6490 SEM (JEOL Instruments, Japan) for these tests. Both setups employed displacement control with a 150 µm diameter flat punch diamond indenter (Synton‐MDP AG, Switzerland).

The Alemnis indenter features a piezoelectric actuator for precise intrinsic displacement control and a strain gauge‐based load cell. However, the actuation speed was limited to 10 µm/s due to the resonance excitation‐based ringing of the load cell at even higher speeds. To achieve a speed exceeding 10 µm/s, a piezo‐based load cell was used, allowing compression speeds up to 10 mm/s. High‐frequency data acquisition systems with the capability of capturing 1 MSamples/s were essential to capture load and displacement data accurately within these short experiment timescales. Additionally, to address resonance and inertia effects on load data, measurements were taken with and without the sample under identical displacement conditions. The specific load related to the sample, excluding resonance and inertial influences, was determined by subtracting the load measured with the sample from that recorded without it [36].

The LTM‐Cryo setup includes a cold finger cooled by liquid nitrogen from an external dewar [37]. Accordingly, the mechanical testing temperature was about −160°C. This cold finger, connected to the tip and sample via copper braids and insulated from the indenter frame by ceramic shafts, features individual resistive heaters and thermocouples. These components, within a closed feedback loop, allow for precise temperature control. To minimize drift, the frame was kept at a constant temperature, and once the system reached equilibrium, the frame heaters were stabilized, ensuring a temperature change of less than ±0.01°C over 10 min. Prior to testing microarchitectures, we synchronized the indenter and sample temperatures to reduce drift, following the previously reported methodology [38]. This involved multiple indentations under load control, adjusting the temperature of either the indenter or sample by ±5°C, and measuring drift during unloading. The optimal temperature pair, resulting in less than 100 pm/s drift, was selected for each test series. At each temperature and strain rate condition, at least 2 microcylinders were compressed.

### 4.5 Finite Element Analysis

This study conducted a mechanical finite element (FE) analysis to simulate the compression response of Cu cylinders using the Johnson–Cook plasticity model (Equation (3)), which was widely employed to describe strain‐rate‐ and temperature‐dependent strain hardening behavior.

(3) $$\bar{\sigma }=\left[A+B{\left({\bar{\varepsilon }}^{p}\right)}^{n}\right]\left[1+C\;In\left(\frac{{\dot{\bar{\varepsilon }}}^{p}}{{\dot{\varepsilon }}^{p}}\right)\right]\left[1+{\left|\frac{T-T_{r}}{T_{m}-T_{r}}\right|}^{m}\right]$$

Water was modeled as a nearly incompressible medium with a bulk modulus of 2.2 GPa. Ice was assumed to exhibit perfectly plastic behavior because catastrophic shear fracture of ice was mechanically suppressed under strong confinement by the copper wall. Under such confinement, ice can sustain continued load transfer without abrupt failure, leading to an apparent plastic‐like response. Because true strain hardening of ice was not expected under these conditions, a perfectly plastic model was adopted as a phenomenological representation of fracture suppression rather than intrinsic plasticity. The detailed definitions of all model parameters were provided in the corresponding Table 1. The hardening parameters of Cu (*A, B, n*) and the thermal softening exponent (*m*) were calibrated through a genetic‐algorithm‐based optimization procedure using the experimental load–depth data from the empty Cu cylinders tested at room temperature and cryogenic conditions. Other Cu parameters—including elastic modulus, Poisson's ratio, and strain‐rate and temperature coefficients—along with the material properties of water and ice, were assigned based on established values reported in the literature (see Table 1). The yield stress of ice, however, was additionally optimized using experimental measurements to ensure accurate representation of its mechanical response.

**TABLE 1:** Materials’ properties for finite element analysis.

Copper (Johnson‐Cook model)		
Young's modulus	140	GPa
Poisson's ratio	0.34	1
*Yield strength (A)	232	MPa
*Strength coefficient (B)	265	MPa
*Hardening exponent (n)	0.56	1
Strain rate strength coefficient	0.025	1
Reference strain rate (${\dot{\varepsilon }}_{0}$)	0.01	1/s
Reference temperature (T_r_)	25	°C
Melting temperature (T_m_)	1085	°C
*Thermal softening coefficient (m)	0.62	1
Water		
Density	1000	kg/m^3^
Bulk modulus	2.2	GPa
Ice (Elastic‐perfect plastic)		
Young's modulus	9	GPa
Poisson's ratio	0.32	
Yield strength	300	MPa

The FE analysis was performed implicitly using the commercial software ABAQUS. For cylinder compression, the geometric dimensions of the specimen model were the same as the CAD model in Figure 1a. A 3D eight‐node linear hexahedral element with reduced integration (C3D8R) was used to mesh the specimen, water, and ice, all modeled with homogenized isotropic properties. The top and bottom plates were defined as rigid bodies; the bottom plate was fully constrained, while displacement control was applied to the top plate. Under this loading condition, the water‐filled Cu and room‐temperature empty Cu cylinders were compressed up to 12.9 µm, whereas the ice‐filled Cu and cryogenic empty Cu cylinders were compressed up to 16.5 µm. Surface‐to‐surface contact was assigned between the specimen and the two plates, as well as between the ice and specimen surfaces. A mechanical hard‐contact formulation was imposed in the normal direction, and a tangential friction coefficient of 0.1 was applied. The interaction between the water and the inner Cu cylinder was modeled using the hydraulic fluid cavity approach, which was commonly employed in structural analyses to capture the behavior of fluids confined within a cavity, such as in hydraulic cylinders.

Additional FE simulations were conducted to validate the deformation mode of the push‐to‐pull geometry and to assess the mechanical contribution of encapsulated ice at cryogenic temperatures. Copper was modeled using the calibrated Johnson–Cook plasticity law, while ice was assumed to follow an elastic–perfectly plastic constitutive relation. First, a full 3D model of the structure (C3D10 elements) was compressed to 20 µm. Because the copper–ice interface includes groove‐like features that led to convergence issues, the copper–ice region was temporarily modeled as a continuous domain with material‐property assignment (copper vs. ice) without explicit contact separation.

Tension simulation of the gauge section of the push‐to‐pull structure was performed using COMSOL Multiphysics 6.0. For simplification of the push‐to‐pull architecture, only the region undergoing tensile deformation, which contains ice, was modeled. A 2‐D axisymmetric model was employed, and the geometry used for the simulation is shown in Figure S15. The ice‐encapsulated cylindrical region was modeled using the actual geometry, and the top and bottom plates of the cylindrical structure were assigned a diameter of 28 µm to match the cross‐sectional area of the rectangular part of the experimental specimen. Two types of copper–ice interfaces were considered to account for surface roughness originating from the voxel‐by‐voxel printing process. One interface was assumed to be perfectly smooth, while the other incorporated geometric roughness. The rough surface was modeled using a sinusoidal profile with an amplitude of 0.25 µm and a wavelength of approximately 2.1 µm. Contact interactions were modeled using the augmented Lagrangian formulation implemented in COMSOL. All simulations were conducted under static conditions, corresponding to material behavior evaluated at the reference strain rate in the Johnson–Cook formulation. Tensile loading was applied by prescribing equal and opposite displacements of 1 µm at the top and bottom surfaces, resulting in a total tensile displacement of 2 µm.

To ensure the numerical accuracy and convergence of the FE simulations, a systematic mesh refinement study was conducted until the mesh configuration yielded negligible changes in the predicted mechanical response. The mesh density was progressively increased by reducing the characteristic element size, while all material properties, boundary conditions, and loading conditions were kept identical across simulations.

The present FE simulations were limited to a strain rate of 0.001 s^−^^1^. While this rate captures the baseline temperature dependence essential for interpreting Figures 2 and 3, future work will extend the numerical model to additional strain rates to more comprehensively examine rate–temperature coupling in confined liquid/ice systems.

## Conflicts of Interest

The authors declare no conflicts of interest.

## Supporting information




**Supporting File 1**: adma72435‐sup‐0001‐SuppMat.docx.

**Supporting File 2**: adma72435‐sup‐0002‐MovieS1.avi.

**Supporting File 3**: adma72435‐sup‐0003‐MovieS2.mp4.

**Supporting File 4**: adma72435‐sup‐0004‐MovieS3.mp4.

**Supporting File 5**: adma72435‐sup‐0005‐MovieS4.mp4.

**Supporting File 6**: adma72435‐sup‐0006‐MovieS5.mp4.

**Supporting File 7**: adma72435‐sup‐0007‐MovieS6.mp4.

**Supporting File 8**: adma72435‐sup‐0008‐MovieS7.mp4.

**Supporting File 9**: adma72435‐sup‐0009‐MovieS8.mp4.

**Supporting File 10**: adma72435‐sup‐0010‐MovieS9.mp4.

## Data Availability

The data that support the findings of this study are available from the corresponding author upon reasonable request.
