# Supplementary material for: Thermomechanics of Picoliter Liquids Encapsulated in Metal Microarchitectures
Source: Adv Mater. 2026 Feb 27;38(17):e15677. doi: 10.1002/adma.202515677 (PMC13003907; doi:10.1002/adma.202515677)
Supplement: Supplementary file 1 — Supporting File 1: adma72435‐sup‐0001‐SuppMat.docx. [file ADMA-38-e15677-s004.docx]

**Supporting Information**

Thermomechanics of picoliter liquids
encapsulated in metal microarchitectures

Sung-Gyu Kang ^a, b†^*, Kyeongjae Jeong ^c†^, Bárbara Bellón ^a^, Lalith Kumar Bhaskar ^a^, Leonardo Shoji Aota ^a^, Jeongin Paeng ^a^, Dipali Sonawane ^a^, Kuan Ding ^a^, Se-Ho Kim ^d^, Allison Goetz ^e^, Benjamin Apeleo Zubiri ^e^, Erdmann Spiecker ^e^, Ayman El-Zoka ^f^, Baptiste Gault ^a, f, g^, Gerhard Dehm ^a^, and Rajaprakash Ramachandramoorthy ^a, h^ *

^a^ Max-Planck-Institute for Sustainable Materials, Max-Planck-Straße 1, 40237 Düsseldorf, Germany

^b^ Department of Materials Engineering and Convergence Technology, Gyeongsang National University, Jinju-daero 501, 52828 Jinju, Republic of Korea

^c^ School of Advanced Materials Science and Engineering, Sungkyunkwan University, Seobu-ro 2066, 16419 Suwon, Republic of Korea

^d^ Department of Materials Science and Engineering, Korea University, Anam-ro 145, 02841 Seoul, Republic of Korea

^e^ Department of Materials Science and Engineering, Institute of Micro- and Nanostructure Research, and Center for Nanoanalysis and Electron Microscopy (CENEM), Friedrich-Alexander-Universitat Erlangen-Nürnberg, IZNF, Cauerstraße 3, 91058 Erlangen, Germany

^f^ Department of Materials, Royal School of Mines, Imperial College London, Prince Consort Road, SW7 2BP London, United Kingdom

^g^ Present address: Univ Rouen Normandie, CNRS, INSA Rouen Normandie, Groupe de Physique des Matériaux, UMR 6634, F-76000 Rouen, France

^h^ Department of Materials Engineering, KU Leuven, Gebroeders De Smetstraat 1, 9000 Gent, Belgium

^†^ These authors have contributed equally

**Supporting Figures**

**Figure S1.** Step-by-step schematic of liquid-filled copper microcylinder fabrication process. (a-c) During LEL process. (d) As-fabricated microcylinder exposed to air after printing.


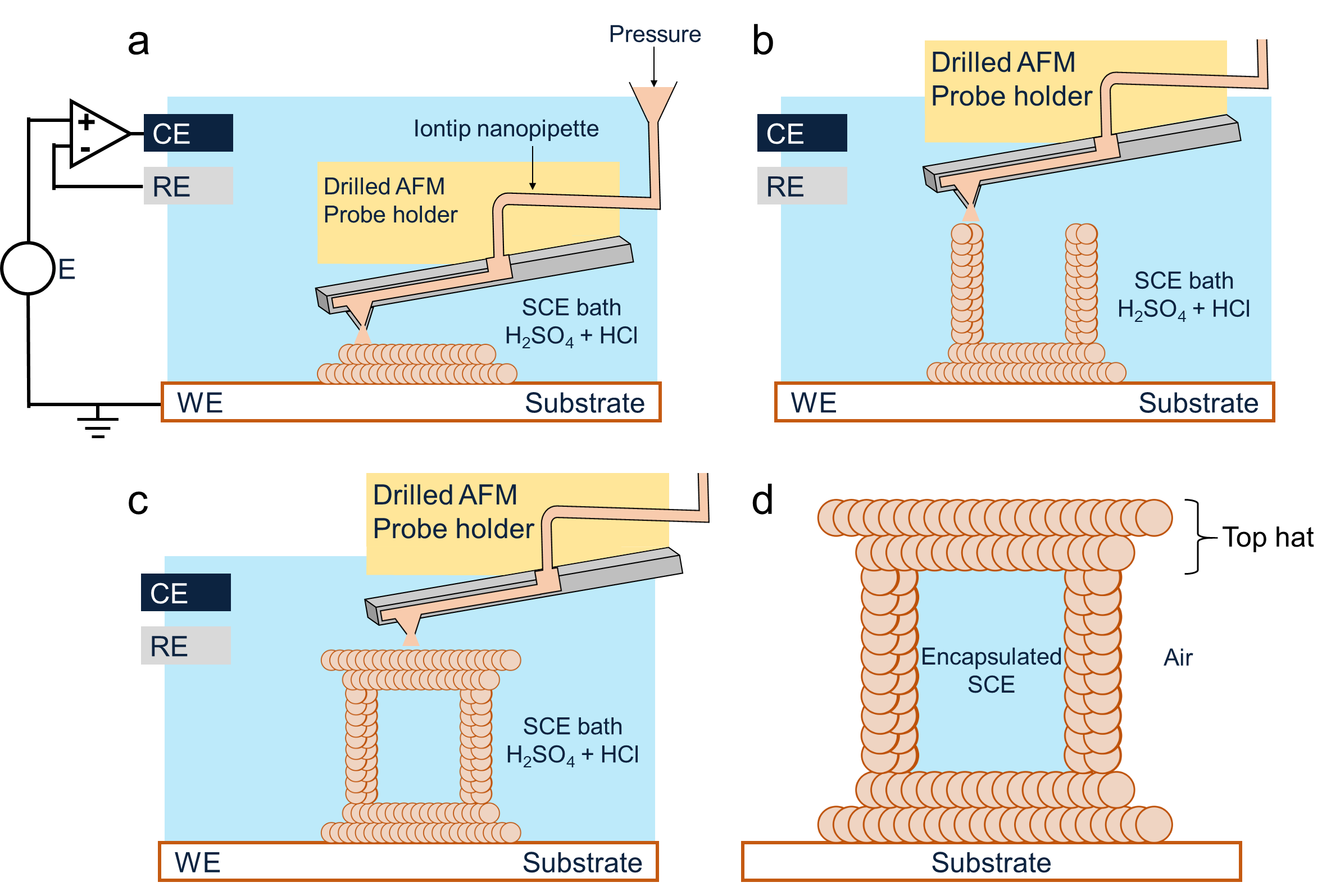


**Figure S2.** X-ray absorption contrast images of copper microcylinder obtained from nanoCT analysis. (a) SEM image of as-fabricated microcylinder showing cross-sections of interest. (b) Cross-sectional X-ray absorption contrast images.


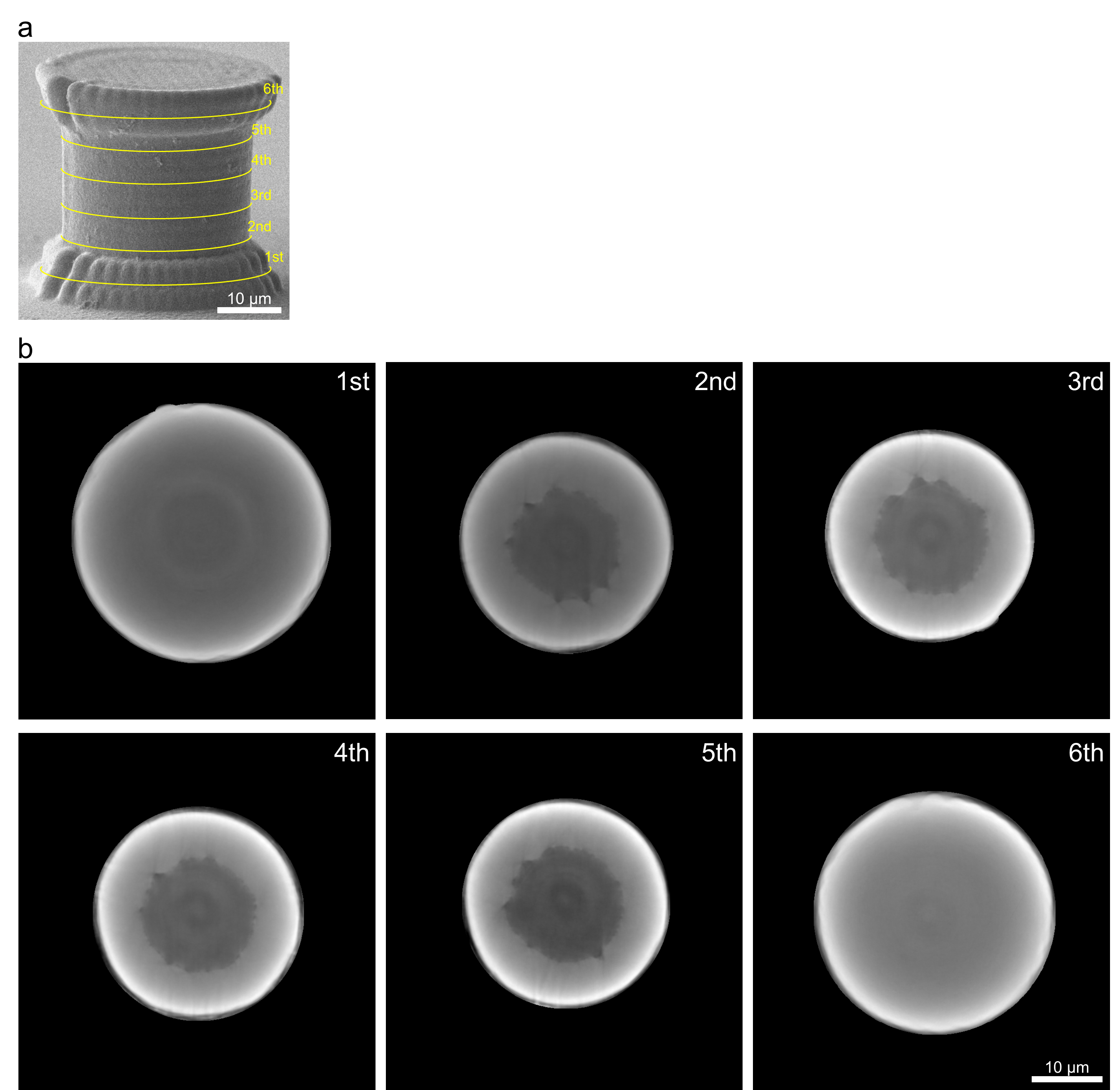


**Figure S3.** Fabrication and *in situ* heating of copper microcylinder without encapsulated liquid. (a-c) Step-by-step schematic of fabrication process. (a, b) During LEL process. (c) As-fabricated microcylinder exposed to air and vacuum of the SEM chamber. (d) Cross-section SEM image of microcylinder showing a pore channel at the bottom plate. SEM images of microcylinder which is (e) as-fabricated and (f) heated to 250 °C, respectively.


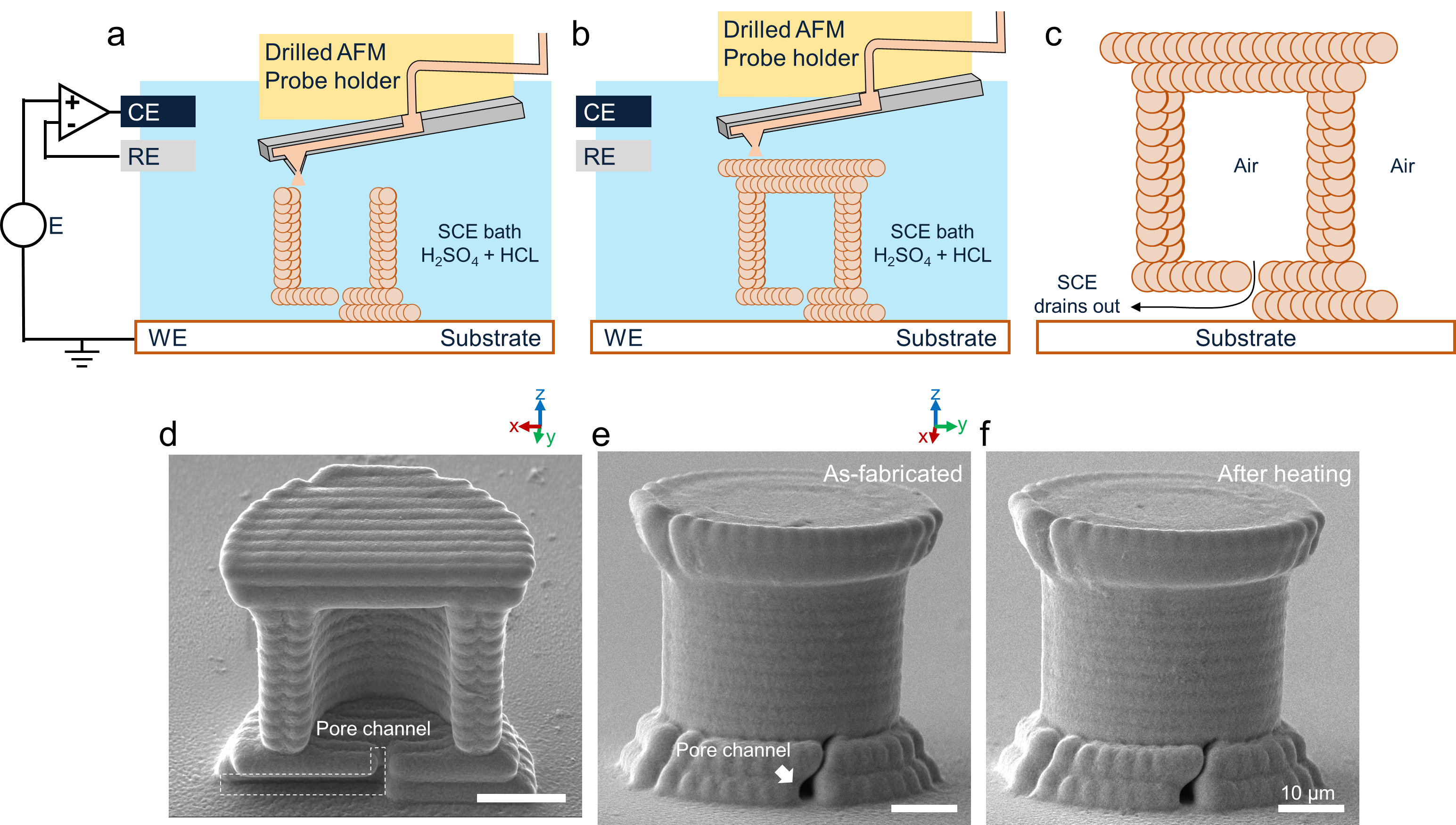


**Figure S4.** APT analysis of the copper wall obtained from liquid encapsulated microcylinders. Elemental distribution of (a) as-fabricated microcylinder and (b) heated microcylinder to 250 °C, respectively. Images 1-3 in (a) indicates pole figures from the detector map, showing three different grains detected along the tip height. (c) Isosurface created from (b). Element concentration line map of (d) Cu and (e) other elements as shown in (c).


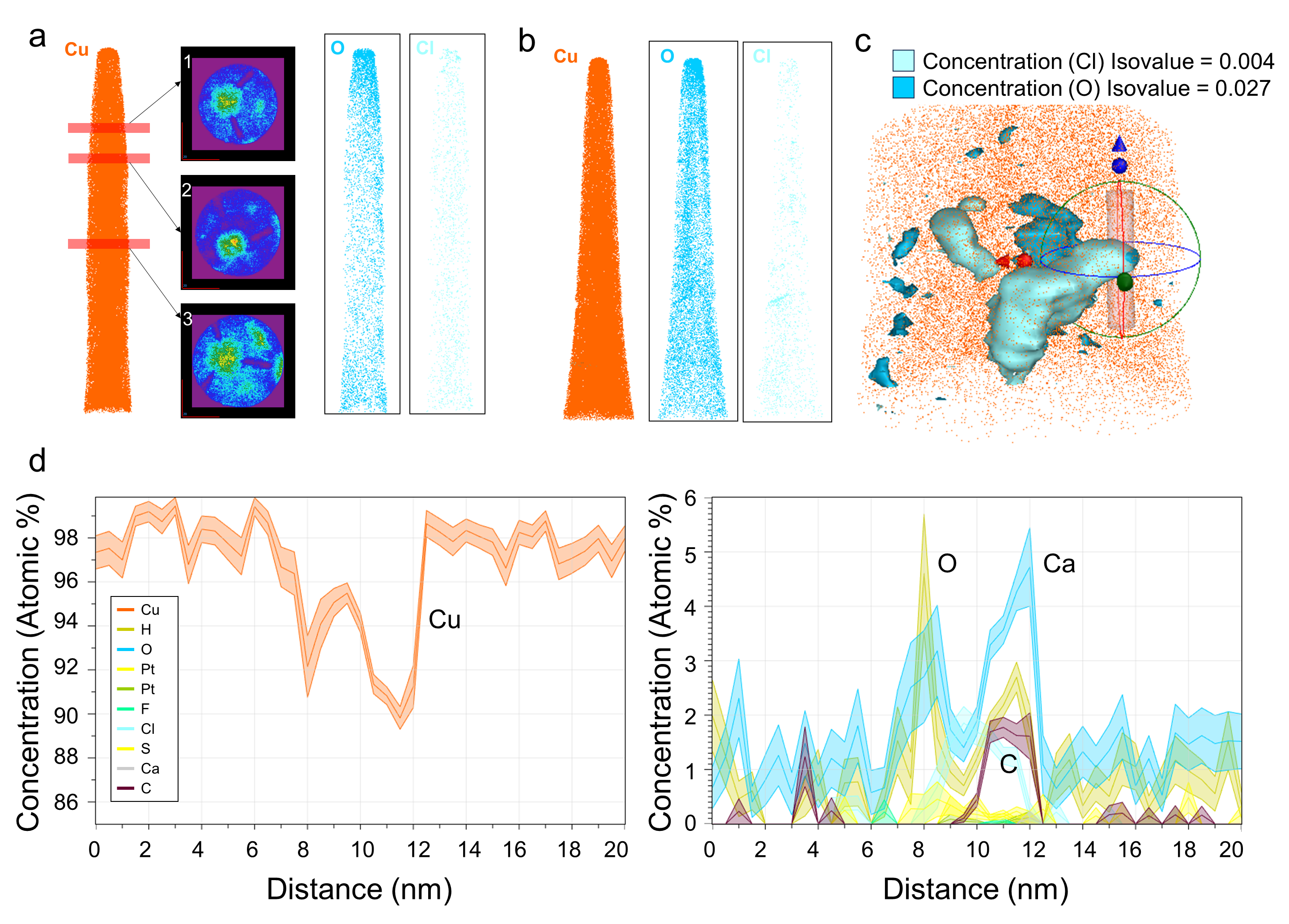


**Figure S5.** Drop strain with respect to strain rate. Drop strain is the strain where stress drop occurs in stress-strain curves of liquid-filled copper microcylinders.


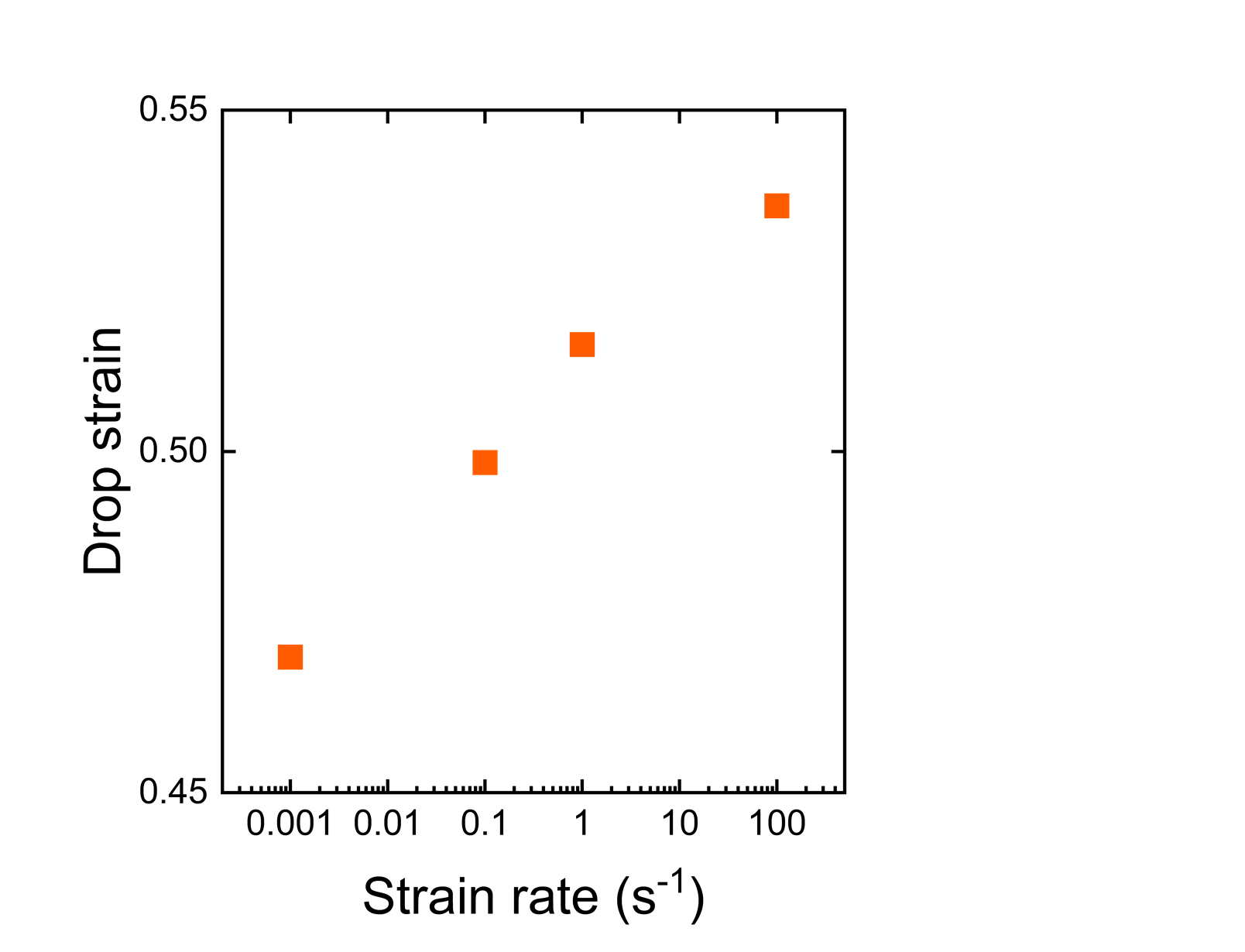


**Figure S6.** Compression load-displacement curves of unfilled copper cylinders at RT and strain rates of 0.001/s and 0.1/s. Scatter plots were obtained from FE simulation with Johnson-Cook parameters.


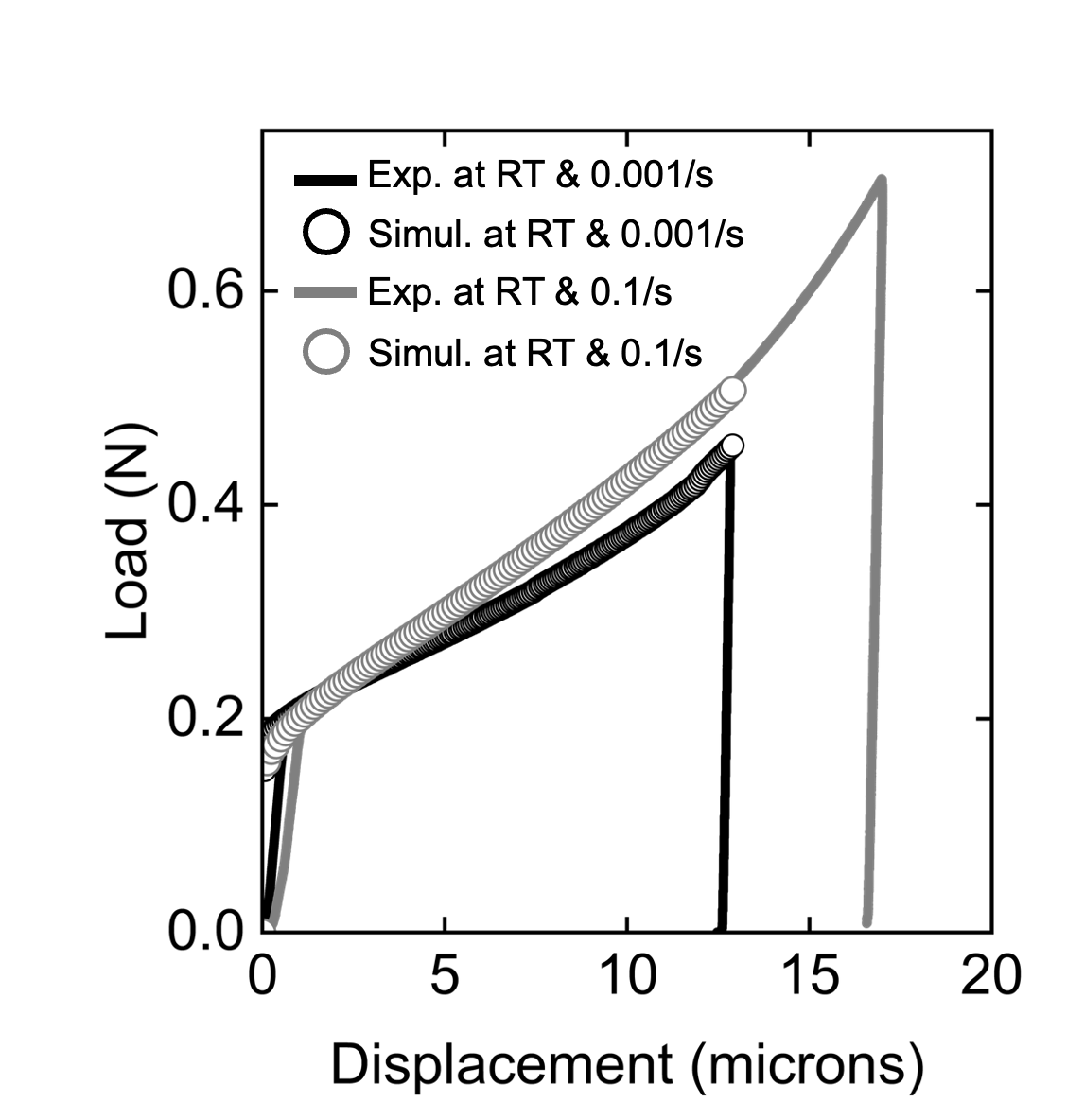


**Figure S7.** Top plate thicknesses of copper microcylinders compressed to $\varepsilon=0.5$ at various strain rates and RT.


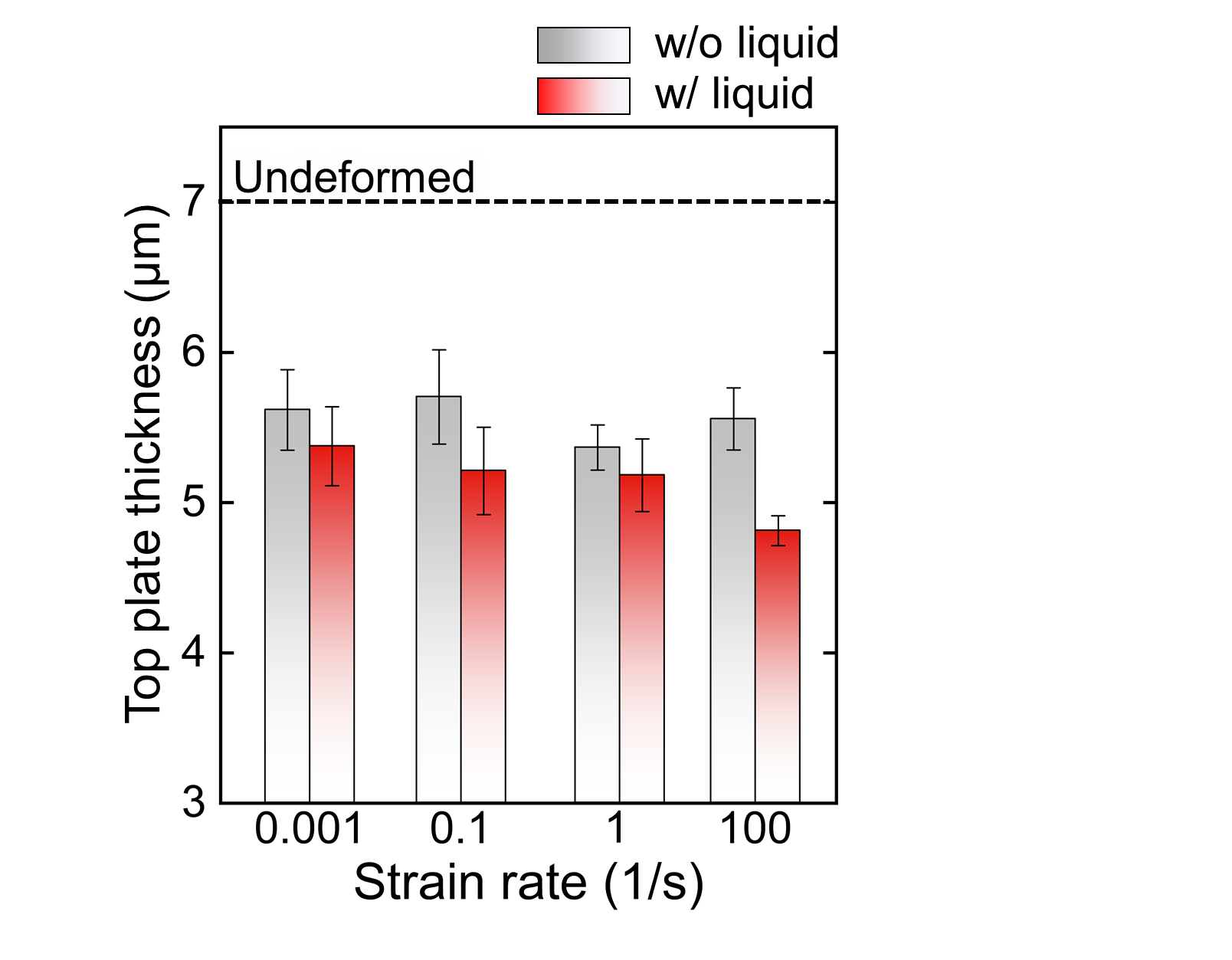


**Figure S8.** Cross-sectional analysis of a microcylinder without encapsulated liquid. (a) SEM image of the cylinder from the top view, with overlaid printing voxel schematic. (b) Cross-sectional SEM image of the FIB-milled cylinder, with a white arrow indicating the printing defect on the top plate. (c) Schematic of the printing step at which the defect in the top plate occurs.


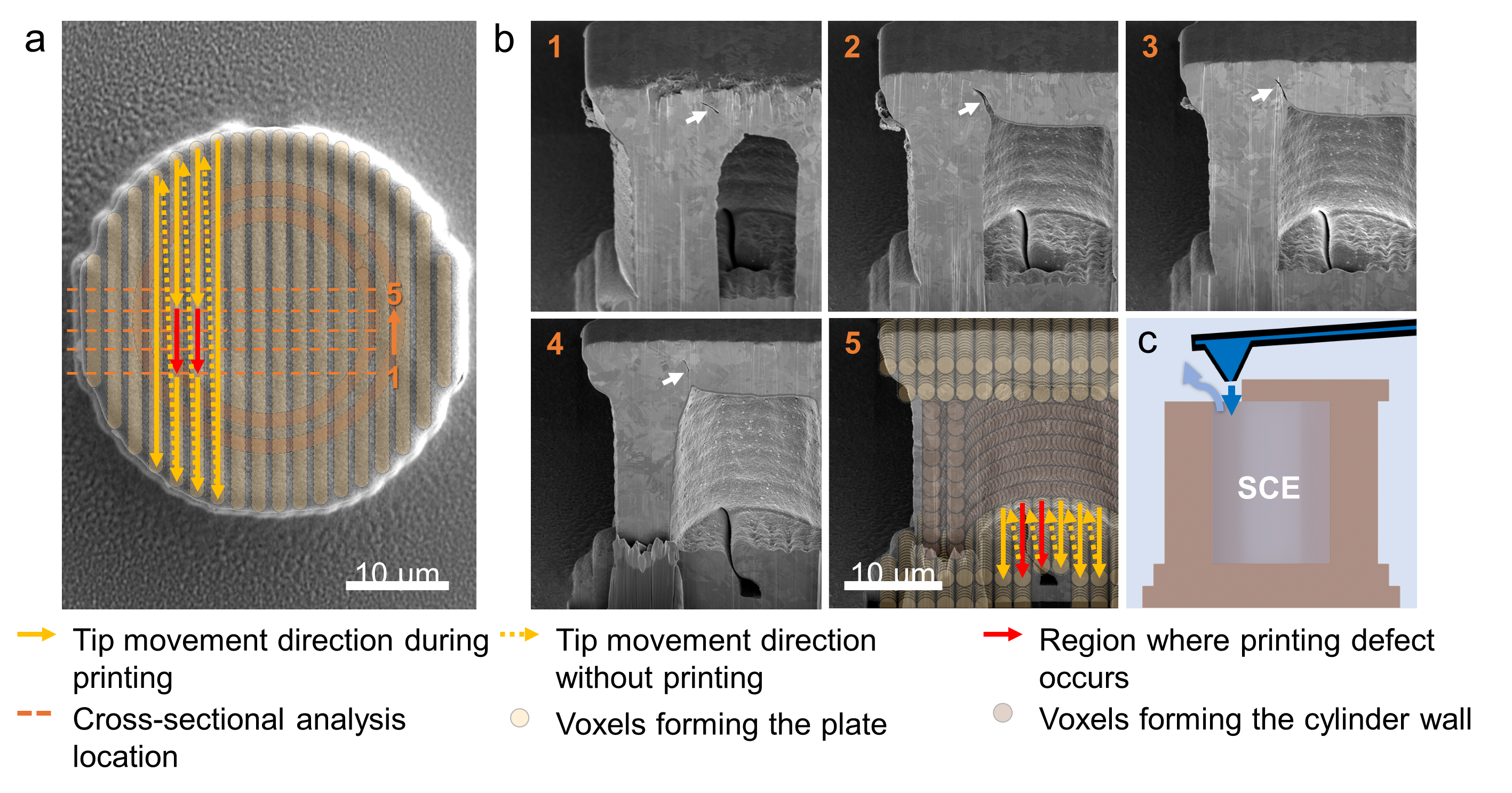


During printing, the tip follows the prescribed voxel sequence defined in the printing file. To enable electrodeposition, ink is released from the tip under pressure, also specified in the file. This implies that for electrodeposition to proceed properly, there must be a continuous liquid flow beneath the tip, not only of the ink but also of the SCE to accompany it. However, when the liquid underneath is semi-confined by surrounding printed structures, it cannot flow away easily. **Figure S8(a)** shows the SEM image of the cylinder from the top view overlaid with the printing voxel schematic and the region of the cross-sectional analysis. From the cross-sectional milling of a microcylinder without encapsulated liquid (**Figure S8(b)**), printing defects can be observed in the top plate and also in the bottom plate, which were not intentional. By examining the printing sequence, we found that these defects correspond to voxels located in later stages of the print among the voxels with no supporting printed structures existing beneath them. In the case of the bottom plate, the defect occurs as the second voxel layer attempts to cover over an intentional hole left by the first layer (see fifth cross-sectional image of **Figure S8(b)**). When pressure is applied to the tip at those voxels, SCE needs to flow away either laterally through the hole or upward along the tip’s side, which is almost the opposite direction of the ink flow. This possible upward flow of SCE may disrupt the electrodeposition process by introducing unwanted flow into the printing region and could also cause tip bending which may disturb proper printing. A similar defect appears in the specific location of the top plate, where the entire cylinder becomes sealed (indicated with red arrows in **Figure S8(a)** and white arrows in **Figure S8(b)**). This defect is located near the bottom side of **Figure S8(a)**, as the space for liquid to escape through the top of the cylinder gradually decreased during printing (see **Figure S8(c)**). These defects are not caused by printing errors such as collisions or skipped voxels, but rather by fluid dynamics, and can therefore be predicted and controlled by adjusting the pressure or routing parameters. Regardless of the printing defect, by printing a second voxel layer in the top plate, the cylinder could be entirely sealed and microstructures with encapsulated liquid could be successfully printed in cylinders without a hole in the bottom plate.

**Figure S9.** Temperature profile of substrate with copper microcylinders during cooling to CT.


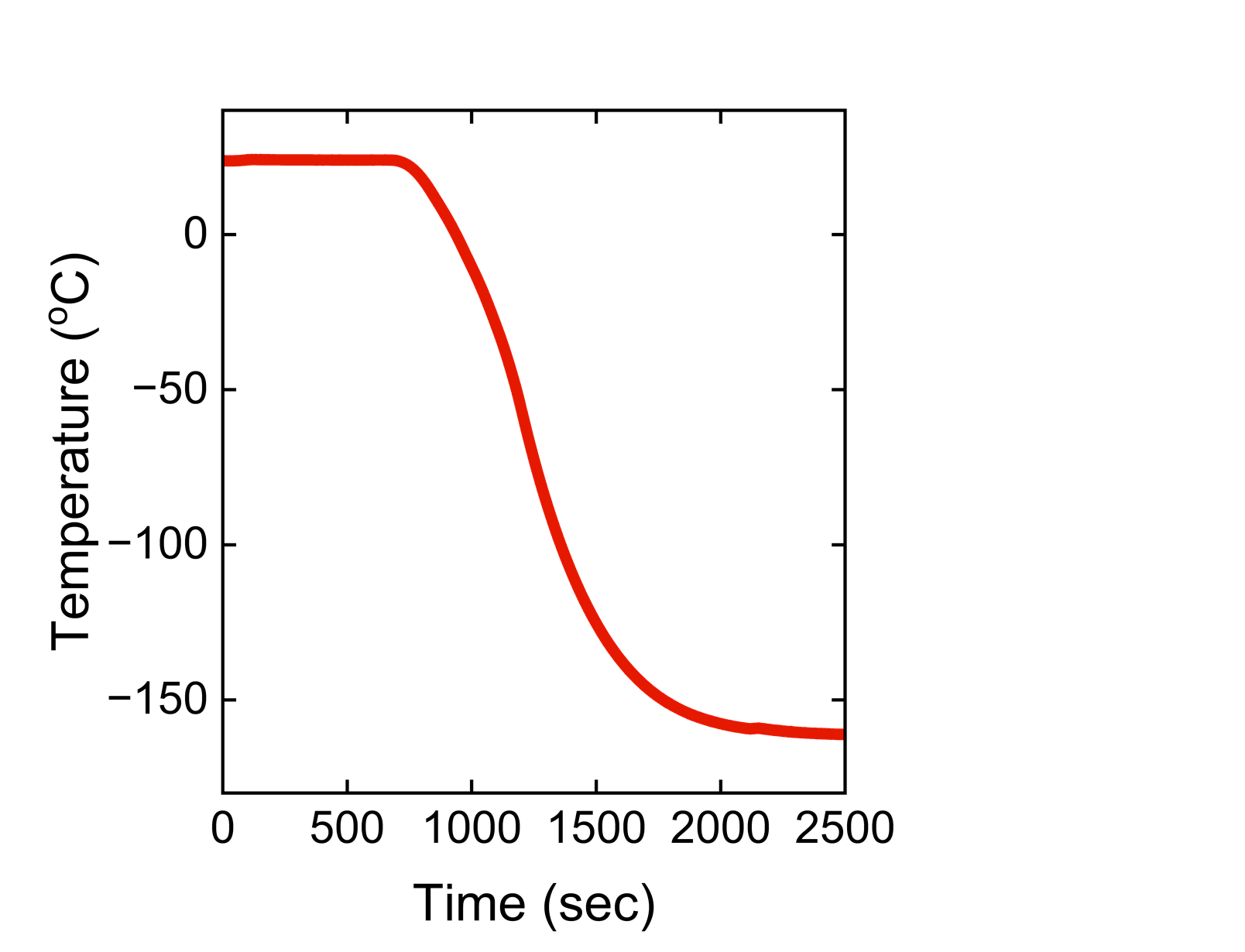


**Figure S10.** Stress and plastic strain distribution of unfilled and liquid-filled microcylinders compressed at CT – assessed using FEM.


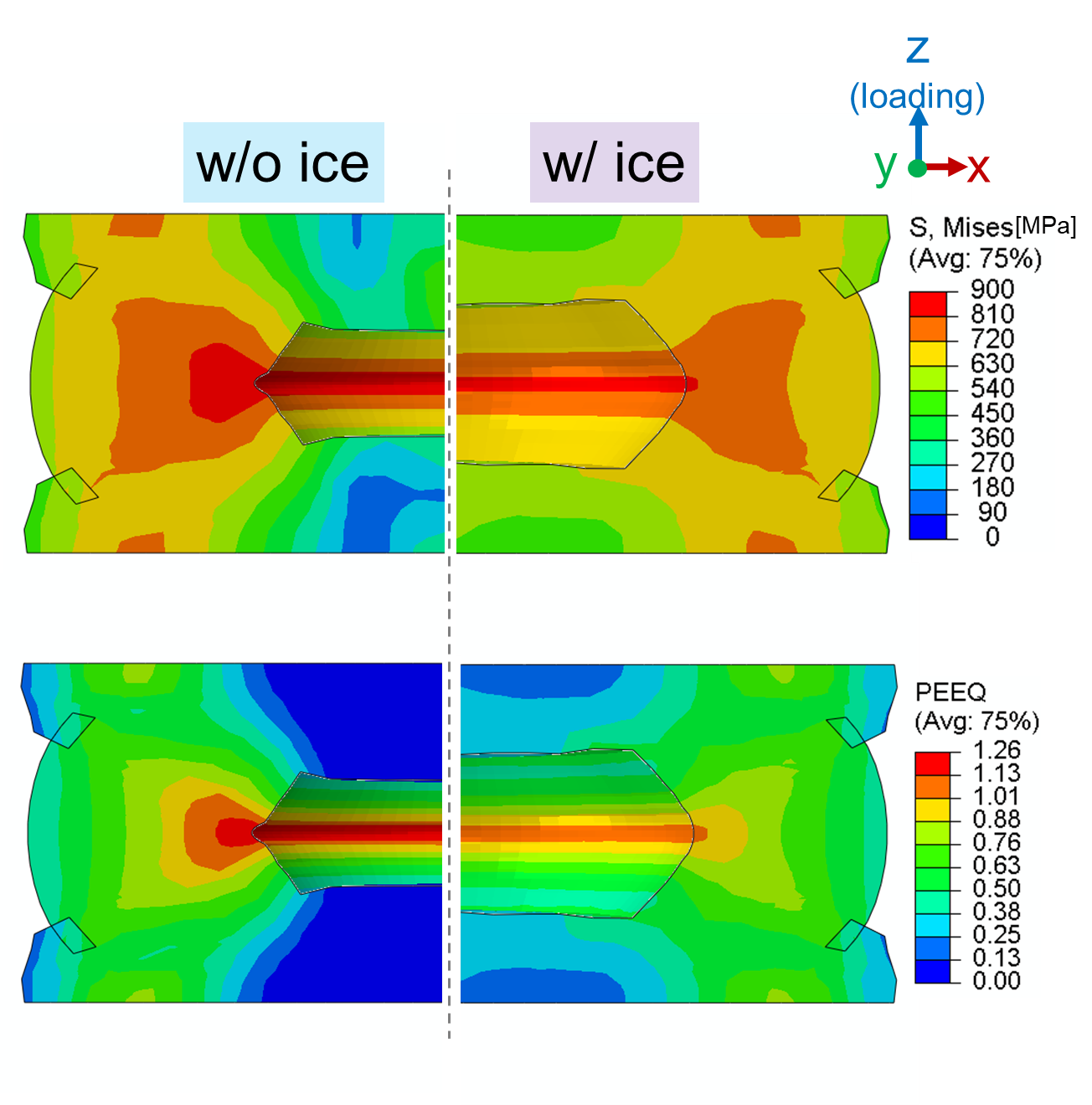


**Figure S11.** Top plate thicknesses of copper microcylinders compressed to $\varepsilon=0.4$ at various strain rates and CT.


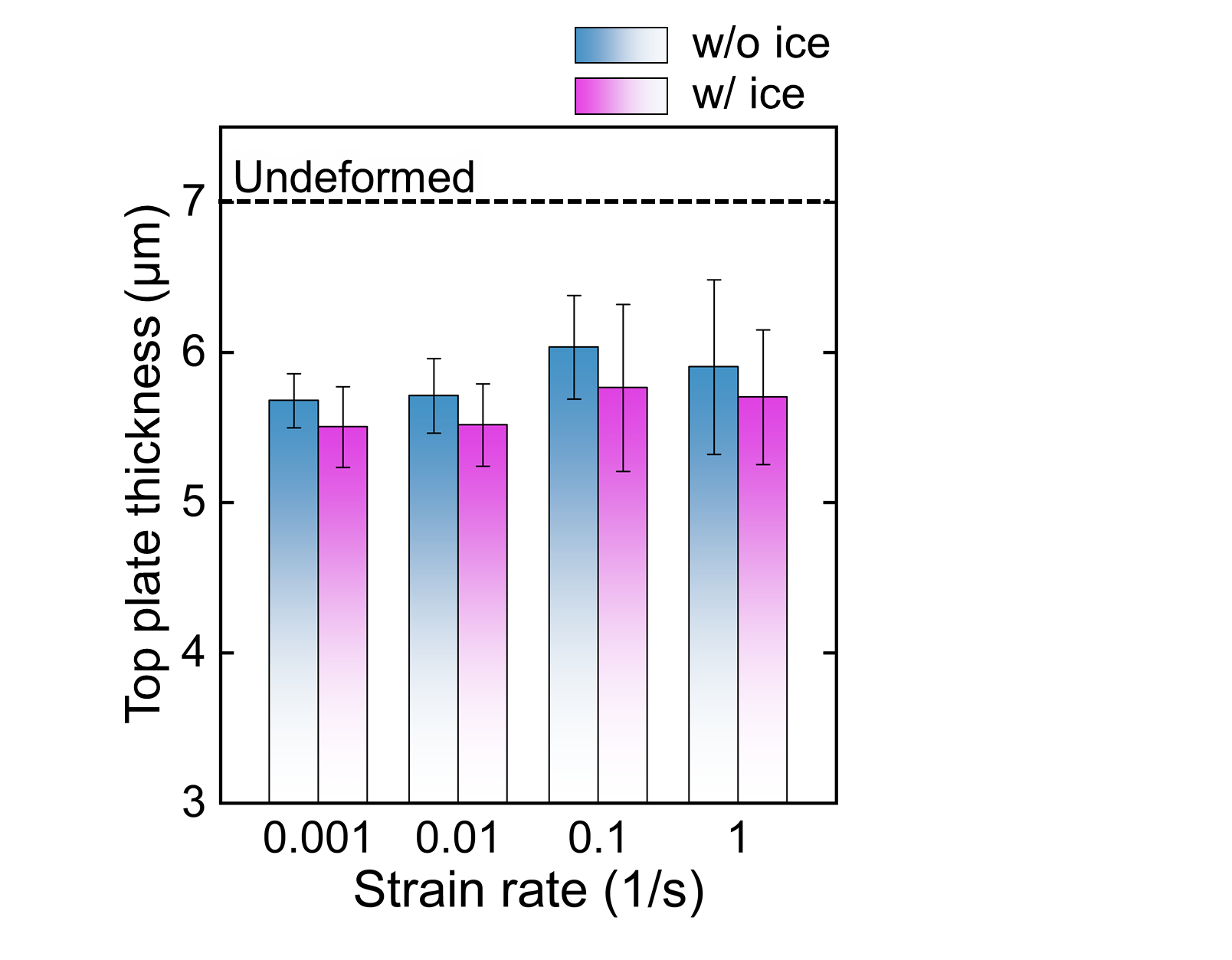


**Figure S12.** Finite element simulation of push-to-pull structure compression. a) Constructed FE model. b) Calculated compression load-displacement curves of push-to-pull structure with and without ice at CT. c-d) Equivalent plastic strain distribution of push-to-pull structures without and with ice, respectively, at compressive displacement of 4 μm.


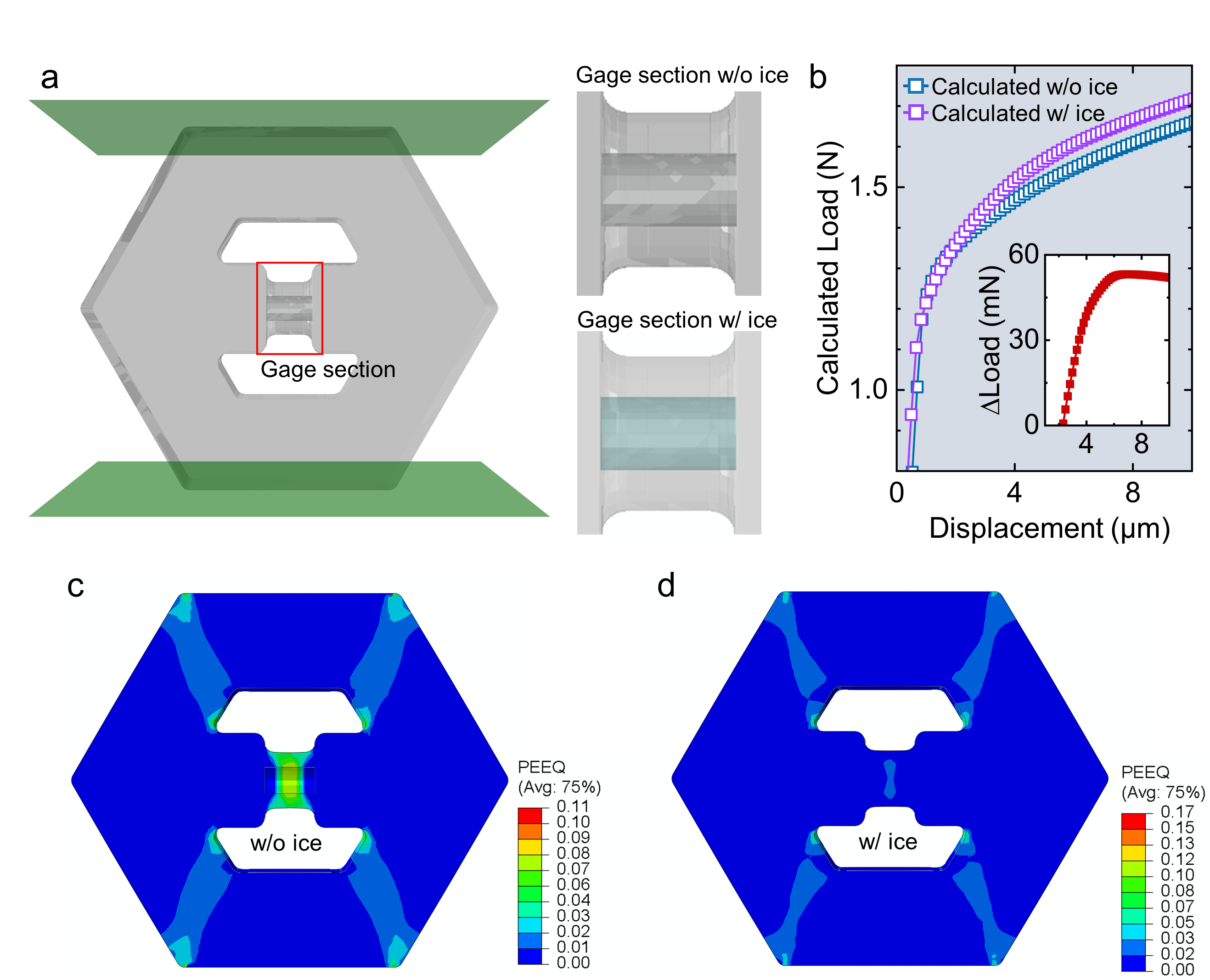


**Figure S13.** Compression test of copper push-to-pull structure without encapsulated ice at cryogenic temperature of -160 °C.


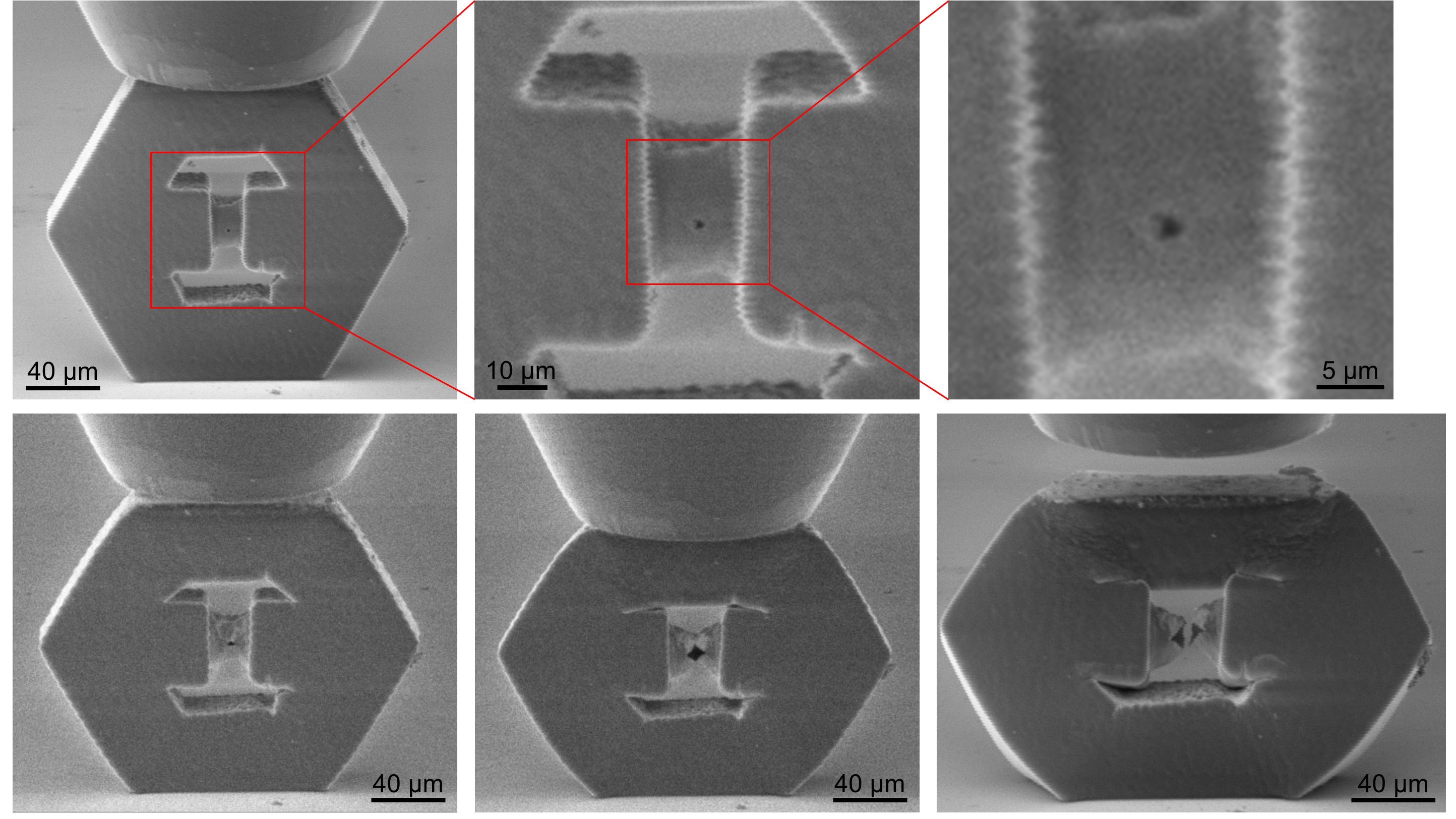


**Figure S14.** Notch-like printing defects at inner surface of copper wall. (a) Cross-section SEM image of as-printed microcylinder. (b) Maximum depth of notch and a half distance between notches.


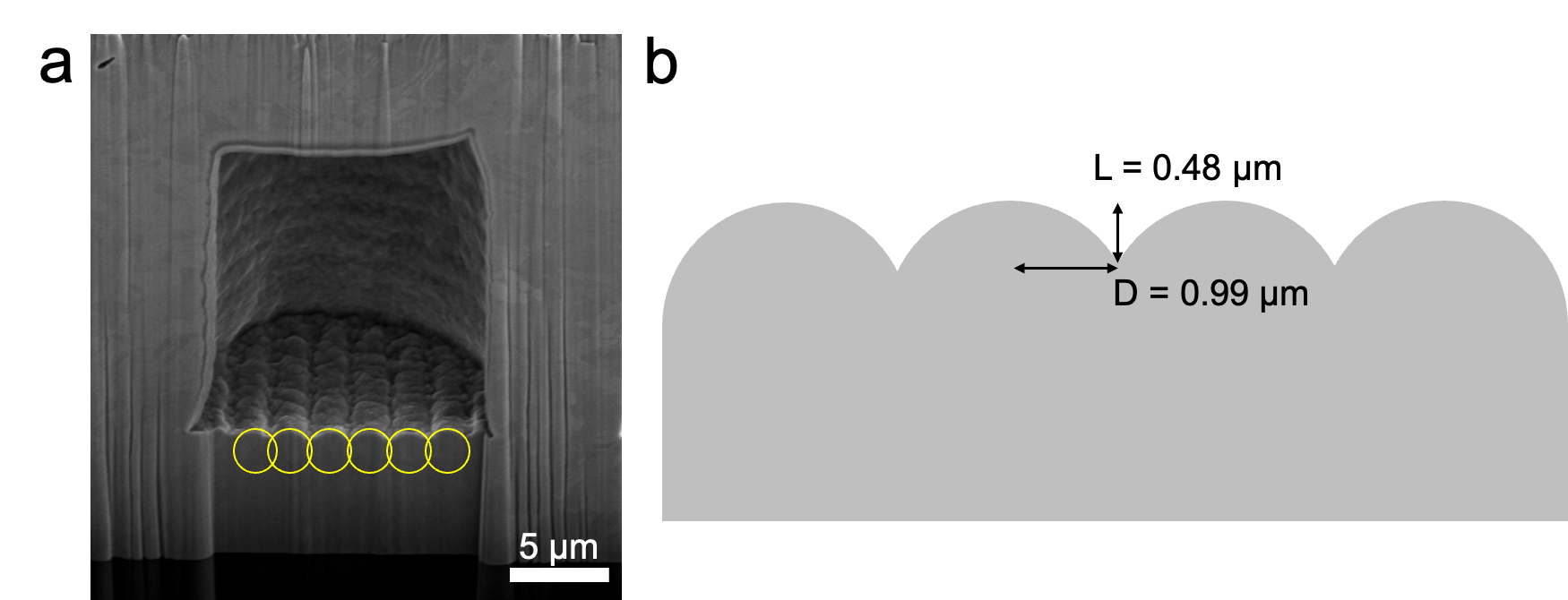


**Figure S15.** Finite element simulation of isolated gauge section deformation of push-to-pull structure. a-b) Constructed axisymmetric FE models with smooth and rough interfaces, respectively. c) Calculated compression load-displacement curves of the gauge section with and without ice at CT. d) Mises stress distribution in the gauge section of push-to-pull structures without and with ice, respectively.


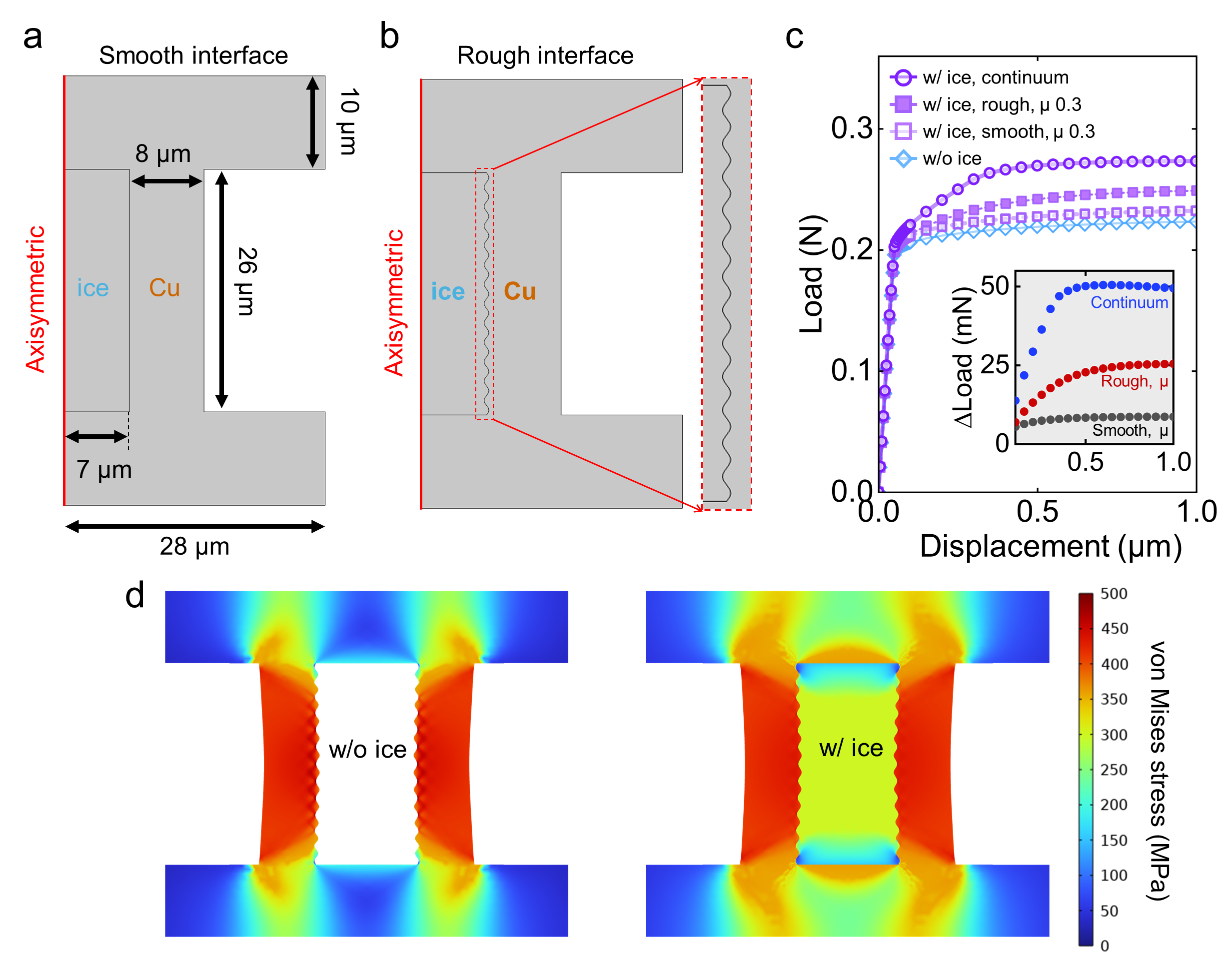


For the smooth interface treated as a continuous domain (no interface friction, just like Model 1), the predicted strengthening is consistent with Model 1, validating the reduced gauge-only approach (ΔL ≈ 50 mN, blue scatter plot in the inset of Figure S15c). Notably, for the smooth interface with friction (μ = 0.3), the existence of ice leads to a negligible strengthening effect (ΔL ≈ 5 mN, black scatter plot in the inset of Figure S15c). However, for the groovy interface with friction (μ = 0.3), the existence of ice leads to a noticeable strengthening effect (ΔL ≈ 25 mN, red scatter plot in the inset of Figure S15c), which is quite close to the experimentally measured difference in load levels of push-to-pull structures with and without ice at yield. For the smooth interface under contact boundary conditions, limited tensile stress is transferred to the ice even in the presence of friction. In contrast, for the rough interface under contact boundary conditions, geometric interlocking combined with friction produces a constraint, leading to effective stress transfer to the ice, and local stress levels could reach the yield strength in parts of the ice region. This result indicates that the experimentally observed strengthening is mechanically consistent with load sharing by confined ice, while also highlighting that the magnitude depends on the roughness of copper–ice interface.
